# D4F‐Functionalized Ceria Nanozyme‐CasRx Platform Suppresses STING and Reprograms the Renal Immune Niche in Acute Kidney Injury

**DOI:** 10.1002/advs.77517

**Published:** 2026-09-06

**Authors:** Weibo Chen, Ming Li, Xuesheng Wang, Jiajia Sun, Ziyuan Zhang, Yongfeng Gong, Benkang Shi, Nengwang Yu, Yunuo Mao, Shengtian Zhao, Xiulin Zhang, Liqiang Guo

**Affiliations:** ^1^ Department of Urology Cheeloo College of Medicine Qilu Hospital Shandong University Jinan China; ^2^ Department of Urology Cheeloo College of Medicine The Second Qilu Hospital of Shandong University Shandong University Jinan China; ^3^ Department of Ultrasound Shandong Provincial Hospital Affiliated to Shandong First Medical University Jinan China; ^4^ Department of Urology Linyi People's Hospital Linyi China; ^5^ Department of Systems Biomedicine School of Basic Medical Sciences Cheeloo College of Medicine Shandong University Jinan Shandong China

**Keywords:** acute kidney injury, CasRx, ceria nanozyme, immune‐niche reprogramming, macrophage‐tubule crosstalk, STING

## Abstract

Acute kidney injury (AKI) is sustained by reciprocal amplification of oxidative stress, innate immune signaling, and maladaptive immune–parenchymal crosstalk. Here, we developed a lipid nanoparticle containing a ceria nanozyme and a STING‐targeting CasRx plasmid and functionalized it with D4F, an 18‐residue apolipoprotein A‐I mimetic peptide composed of D‐amino acids. The resulting D4F‐CeO_2_@LNP‐CasRx(STING) formulation retained radical‐scavenging activity, supported CasRx encapsulation, and showed enhanced cellular and renal delivery. In macrophages, the formulation reduced oxidative stress and total STING abundance together with downstream TBK1 and IRF3 phosphorylation, shifted the CD86/CD206 profile toward a less inflammatory state, and altered the secretome in a manner that protected hypoxia‐challenged tubular epithelial cells. In kidney organoids and peripheral‐blood‐mononuclear‐cell‐coupled organoid cultures, D4F functionalization improved intratissue accumulation, increased the frequency of CD4^+^FOXP3^+^ T cells, and attenuated the hypoxia‐associated DNA damage response and tubular injury. In mice with renal ischemia–reperfusion injury, treatment reduced STING signaling in CD11b^+^ renal macrophages, improved renal function and histology, and lowered whole‐kidney inflammatory and injury readouts. Spatial transcriptomic and proteomic analyses were consistent with contraction of inflammatory immune states and restoration of repair‐associated programs. These findings support a DNA‐delivering nanozyme strategy for renal immune‐niche modulation in AKI.

## Graphical abstract

D4F‐functionalized ceria nanozyme–CasRx nanoparticles preferentially target renal macrophages through SR‐B1‐mediated uptake in ischemia–reperfusion injury. Ceria scavenges excessive ROS, while CasRx silences STING signaling, shifting macrophages toward a less inflammatory, pro‐resolving state and increasing the proportion of regulatory T cells. This coordinated redox and immune modulation alleviates renal injury and promotes AKI recovery.

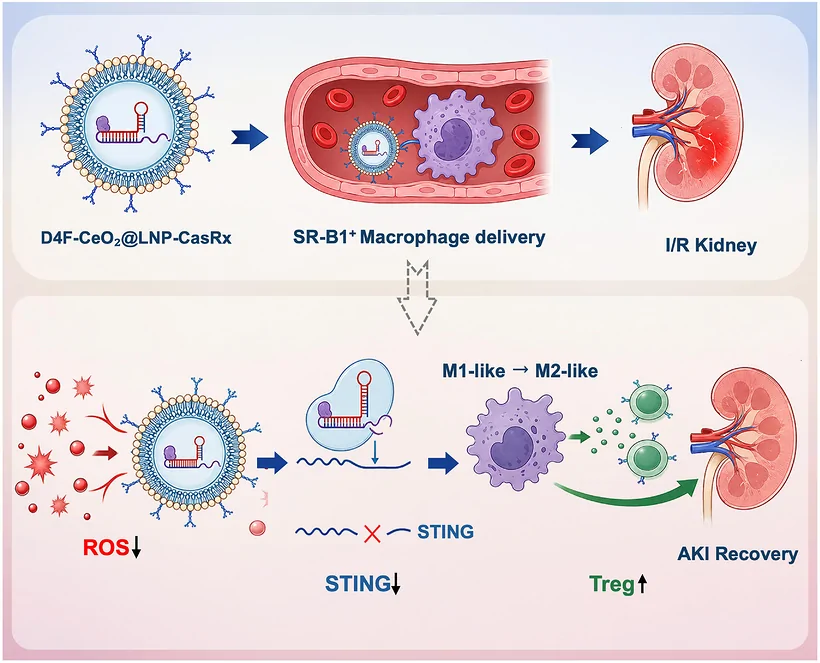

## 1 Introduction

Acute kidney injury (AKI) remains a common and clinically devastating syndrome with high short‐term morbidity and mortality and a substantial risk of later progression to chronic kidney disease [1, 2]. Ischemia‐reperfusion (I/R)‐associated AKI is particularly relevant in surgery, transplantation, shock, and critical care, yet treatment remains largely supportive because the disease is driven by coupled metabolic and inflammatory injury rather than a single dominant lesion [1]. Proximal tubular epithelial cells are central to this process: ischemic and oxidative damage rapidly induces ROS accumulation, mitochondrial dysfunction, danger‐signal release, and inflammatory transcriptional responses, while mitochondrial DNA (mtDNA)‐ and STING‐associated signaling further amplifies tissue injury [3, 4].

At the same time, AKI is increasingly recognized as a disorder of the renal immune microenvironment rather than a purely tubular cell‐autonomous event [5, 6]. Macrophage–tubule interactions, immune‐cell heterogeneity, and maladaptive repair programs strongly influence whether the injured kidney resolves inflammation or progresses toward chronic remodeling [7, 8]. These features suggest that effective therapy must do more than acutely protect tubules; it must also interrupt the reciprocal amplification between oxidative stress and innate immunity while shifting the local injury milieu toward repair [9, 10].

Nanomedicine is well suited to this challenge because nanosystems can combine catalytic activity, nucleic acid delivery, and tissue‐selective transport within one platform [11, 12]. Kidney‐targeted nanotherapeutics, biomimetic delivery systems, and kidney‐cell‐specific nanomedicines have shown encouraging activity in experimental AKI, and ceria‐based nanozymes are especially attractive because of their durable ROS‐scavenging capacity [13, 14]. However, most currently reported systems remain centered on direct tubular cytoprotection and intervene primarily through a single dominant modality, such as ROS scavenging or cargo‐based anti‐inflammatory delivery, with more limited attention to macrophage–tubule crosstalk and renal immune‐niche remodeling [11, 14].

**FIGURE 1:**
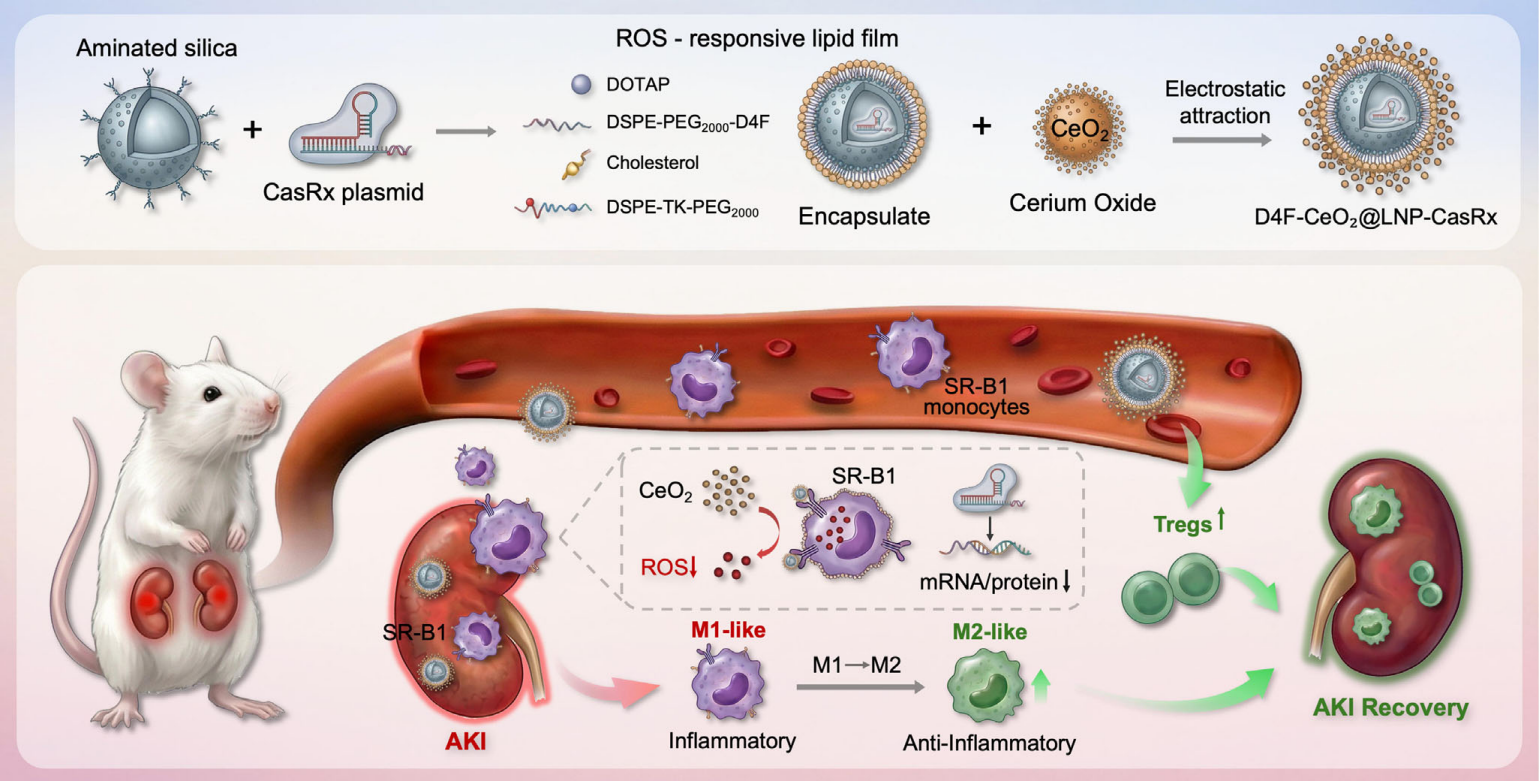
Overview of the D4F‐functionalized ceria nanozyme/CasRx platform. The formulation combines catalytic ROS scavenging with CasRx‐mediated STING transcript depletion and is evaluated for macrophage‐associated renal delivery, modulation of the renal immune environment, and protection from I/R‐AKI.

Programmable RNA targeting offers a route to address this gap. Cas13d/CasRx enables transient transcript‐level silencing without permanent genome editing and is therefore well suited to suppressing inducible inflammatory hubs such as STING [15, 16]. D4F (D‐4F) is an 18‐residue apolipoprotein A‐I mimetic peptide composed of D‐amino acids (DWFKAFYDKVAEKFKEAF) [17]. ApoA‐I mimetic peptides can modulate monocyte/macrophage function and pattern‐recognition responses [17, 18]. We therefore incorporated D4F to promote interaction with the mononuclear phagocyte compartment and engineered D4F‐CeO_2_@LNP‐CasRx(STING) to test whether coordinated redox buffering and RNA‐level STING silencing could protect injured tubules while modulating macrophage–tubule–immune interactions in I/R‐AKI (Figure 1).

## 2 Results

### 2.1 Construction and Characterization of a D4F‐Functionalized Ceria Nanozyme for STING‐Targeted CasRx Delivery

**FIGURE 2:**
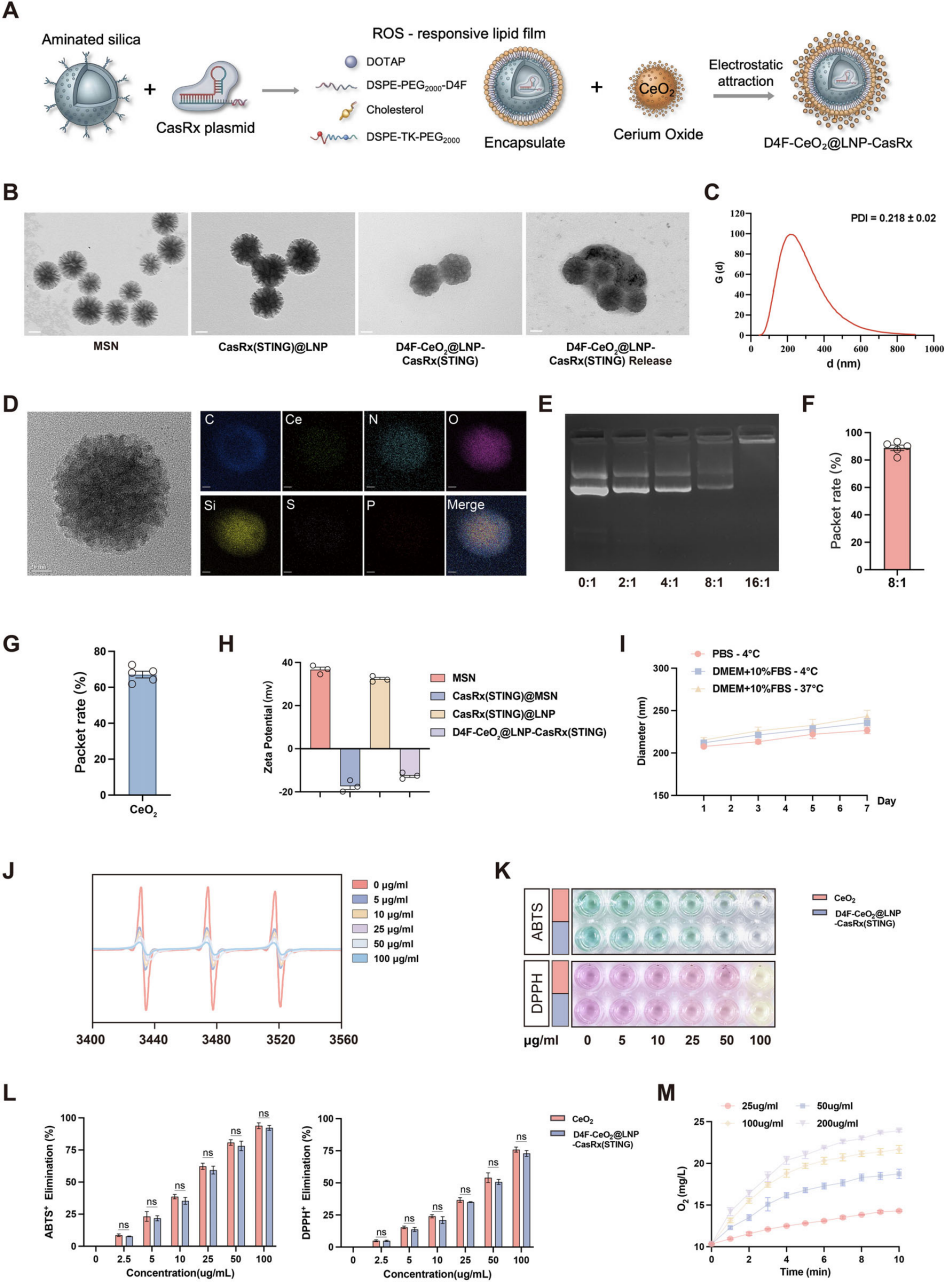
Construction and characterization of D4F‐CeO_2_@LNP‐CasRx(STING). (A) Assembly scheme. (B) Representative transmission electron microscopy (TEM) images of mesoporous silica nanoparticles (MSNs), CasRx(STING)@LNP, D4F‐CeO2@LNP‐CasRx(STING), and D4F‐CeO_2_@LNP‐CasRx(STING) under H_2_O_2_‐triggered release conditions. For brevity, the MSN component is omitted from the labels of the second to fourth images, although all three samples were constructed using MSN as the core scaffold. (C) Hydrodynamic‐size distribution and PDI of the optimized formulation. (D) Elemental mapping. (E) Agarose gel analysis of CasRx loading. (F,G) Packaging efficiencies (*n* = 5). (H) Zeta potential (*n* = 5). (I) Hydrodynamic diameter during 7‐day incubation in PBS at 4°C, DMEM + 10% FBS at 4°C, or DMEM + 10% FBS at 37°C (*n* = 3). (J) ESR spectra at the indicated CeO_2_‐equivalent concentrations (µg/mL). (K) ABTS•^+^ scavenging at the indicated CeO_2_‐equivalent concentrations (µg/mL). (L) DPPH• scavenging (*n* = 3). (M) Dissolved oxygen generated by the indicated CeO_2_‐equivalent concentrations of D4F‐CeO_2_@LNP‐CasRx(STING) during decomposition of 10 mM H_2_O_2_; dissolved oxygen is reported in mg/L (*n* = 3). Data are mean ± SD.

To establish a multifunctional platform capable of simultaneously controlling oxidative stress and inflammatory signaling, we constructed a lipid‐based nanoplatform that integrates a ceria nanozyme, STING‐targeting CasRx cargo, and D4F surface functionalization (Figure 2A). The CasRx expression cassette is shown in Figure S1, and synthesis of the D4F‐conjugated lipid was verified by the reaction scheme and ^1^H NMR spectrum (Figure S2). Transmission electron microscopy visualized the progressive assembly of the nanoplatform (Figure 2B). Dynamic light scattering showed a narrowly distributed hydrodynamic diameter with a polydispersity index (PDI) of 0.218 for the optimized formulation (Figure 2C), whereas elemental mapping confirmed the expected structural elements (Figure 2D). CasRx loading and formulation optimization yielded high packaging efficiency (Figure 2E–G), and the CeO_2_ standard curve used for quantification is provided in Figure S3. The final particles also showed the expected surface‐charge characteristics (Figure 2H). During 7 days in PBS or DMEM containing 10% FBS at 4°C, or in DMEM containing 10% FBS at 37°C, the formulation showed no abrupt increase in hydrodynamic diameter (Figure 2I). At 37°C, the diameter increased gradually from approximately 215 nm on day 1 to approximately 245 nm on day 7, while the PDI remained approximately 0.22–0.25 and below 0.26 (Figure S4A).

We next investigated whether ceria retained its catalytic antioxidant function after formulation. Electron spin resonance showed marked attenuation of the radical signal in the presence of ceria‐containing nanoparticles (Figure 2J). ABTS and DPPH assays likewise showed CeO_2_‐equivalent concentration‐dependent radical elimination (Figure 2K,L). Dissolved oxygen, measured with a dissolved‐oxygen meter, increased with CeO_2_‐equivalent concentration and reaction time when D4F‐CeO_2_@LNP‐CasRx(STING) was incubated with 10 mM H_2_O_2_ (Figure 2M). After 7 days at 4°C, zeta potential and ABTS•^+^ and DPPH• scavenging activity were not detectably changed from day 1 (Figure S4B–D). The optimized formulation showed acceptable cytocompatibility over the tested working range and minimal hemolysis (Figures S5 and S6). These data support short‐term physicochemical stability under the tested conditions while confirming that antioxidant activity was retained after formulation.

### 2.2 D4F Functionalization Enhances Intracellular Delivery and Potentiates Concurrent Suppression of Oxidative Stress and STING Signaling

**FIGURE 3:**
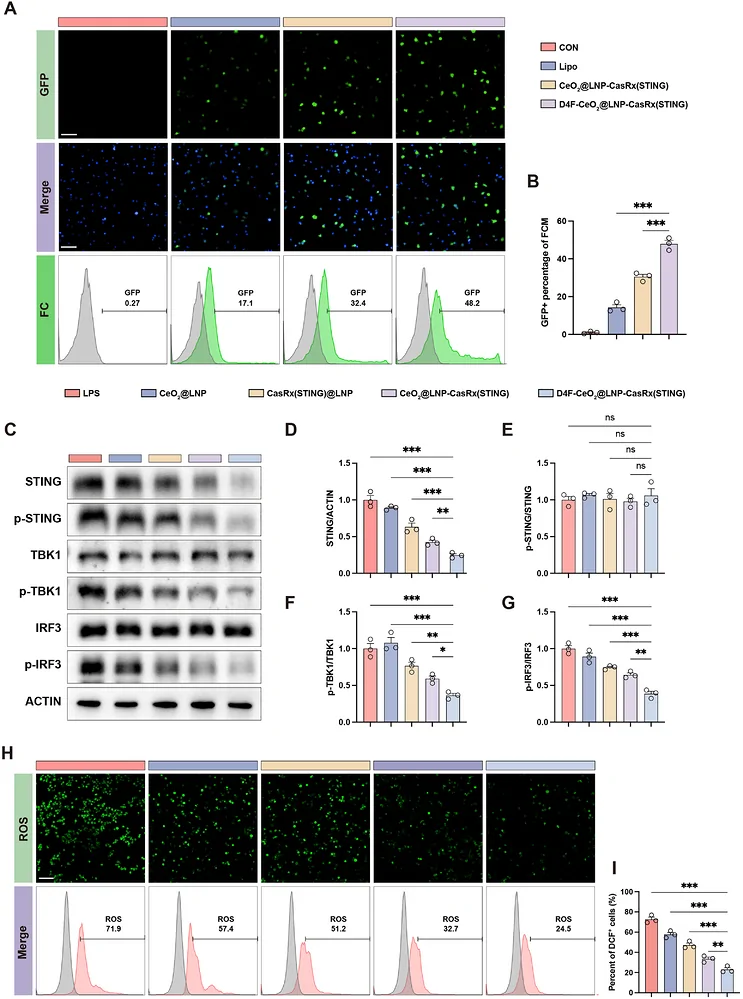
D4F functionalization enhances intracellular delivery and attenuates STING signaling and ROS in LPS‐stimulated RAW264.7 macrophages. (A,B) GFP reporter delivery and GFP‐positive‐cell quantification. (C) Immunoblots of STING, p‐STING, TBK1, p‐TBK1, IRF3, p‐IRF3, and ACTIN. (D–G) STING/ACTIN, p‐STING/STING, p‐TBK1/TBK1, and p‐IRF3/IRF3. (H,I) DCF fluorescence and ROS‐positive‐cell quantification. Scale bar in A and H, 50 µm.Data are mean ± SD (*n* = 3 independent experiments). One‐way ANOVA with Tukey's multiple‐comparisons test; ns, not significant; ^*^ *p* < 0.05, ^**^ *p* < 0.01, and ^***^ *p* < 0.001.

In a GFP reporter experiment, untreated cells showed almost no signal, liposomes alone produced weak GFP expression, CeO_2_@LNP‐CasRx(STING) produced a stronger signal, and the D4F‐functionalized formulation produced the highest GFP‐positive fraction (48.2%; Figure 3A,B). These results indicate that D4F functionalization improves productive delivery in RAW264.7 macrophages.

We next asked whether the gain in intracellular access translated into altered STING signaling. In LPS‐stimulated RAW264.7 macrophages, CasRx‐containing formulations reduced total STING abundance, with the greatest reduction after D4F‐CeO_2_@LNP‐CasRx(STING) treatment (Figure 3C,D). The p‐STING/STING ratio did not differ significantly among the treatment groups (Figure 3E), whereas the p‐TBK1/TBK1 and p‐IRF3/IRF3 ratios decreased progressively and were lowest in the D4F‐functionalized group (Figure 3F,G). Thus, the observed response was defined by reduced STING abundance and attenuation of downstream TBK1 and IRF3 phosphorylation rather than reduced total TBK1 or IRF3 expression.

We therefore tested whether these effects were sequence‐specific using a second independent STING‐targeting guide and a guide‐resistant STING rescue construct. A second, independent STING‐targeting guide recapitulated STING knockdown and the accompanying reduction in TBK1 and IRF3 phosphorylation, whereas guide‐resistant STING partially reversed both effects, supporting sequence‐specific STING silencing as the basis for downstream pathway attenuation (Figure S7).

Because ceria was incorporated to buffer oxidative stress in parallel with CasRx‐mediated STING depletion, we quantified intracellular ROS. LPS increased the DCF‐positive fraction to 71.9%; CeO_2_@LNP and CasRx(STING)@LNP reduced this fraction to 57.4% and 51.2%, respectively; the non‐functionalized dual formulation reduced it to 32.7%, and D4F‐CeO_2_@LNP‐CasRx(STING) reduced it to 24.5% (Figure 3H,I).

### 2.3 The Dual‐Function Nanozyme Shifts Macrophage Activation and Reshapes the Inflammatory Secretome

**FIGURE 4:**
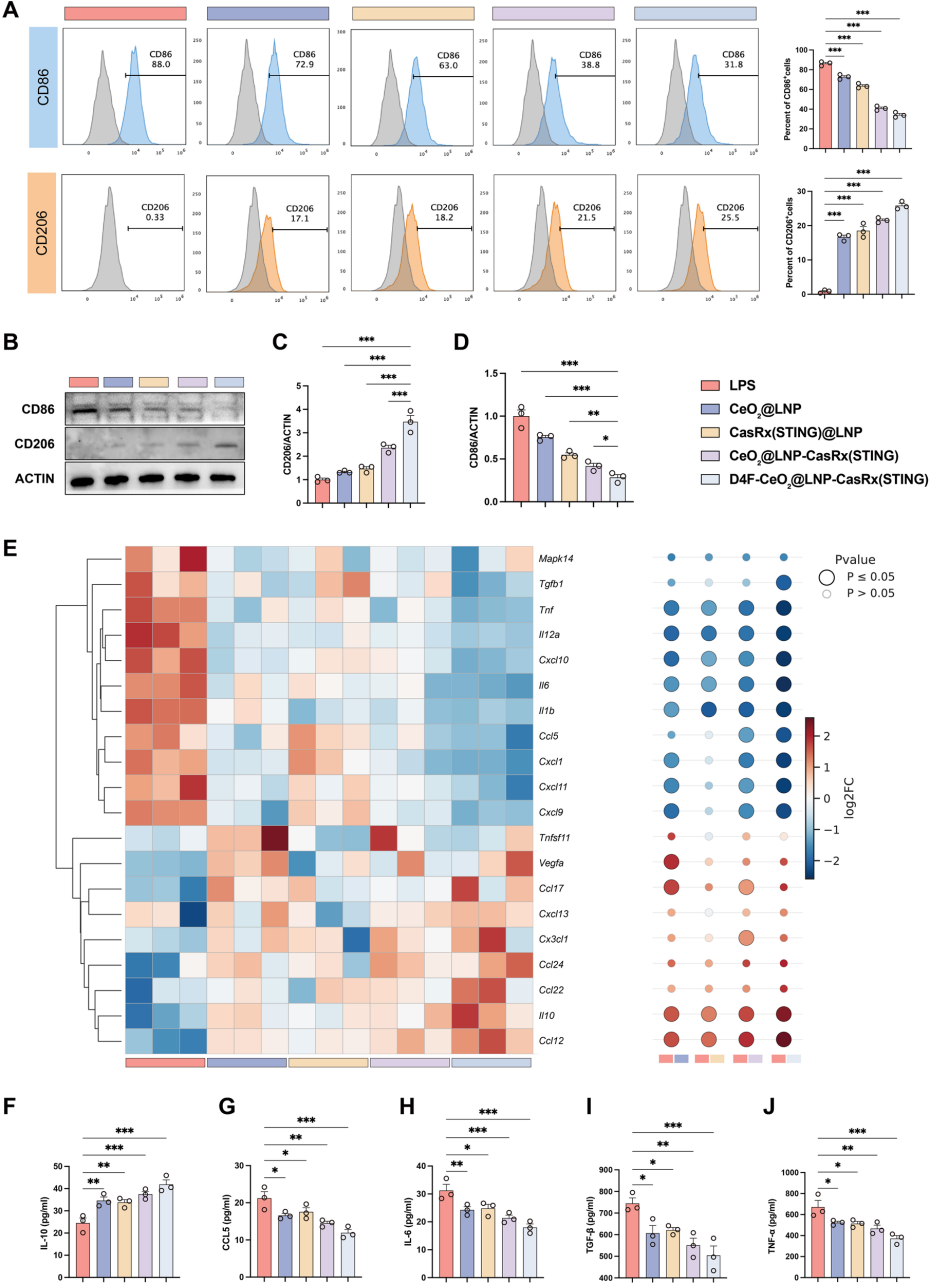
D4F‐CeO_2_@LNP‐CasRx(STING) alters macrophage activation and the inflammatory secretome. (A) CD86 and CD206 flow‐cytometry profiles and quantification. (B–D) Immunoblots and CD206/ACTIN and CD86/ACTIN ratios. (E) qPCR‐array heatmap and bubble plot. (F–J) Secreted IL‐10, CCL5, IL‐6, TGF‐β, and TNF‐α. Panels A and C–J used n = 3 independent biological experiments. Data are mean ± SD; one‐way ANOVA; ns, not significant; ^*^ *p* < 0.05, ^**^ *p* < 0.01, and ^***^ *p* < 0.001.

Because macrophages are central amplifiers of inflammatory injury in AKI, we tested whether D4F‐CeO_2_@LNP‐CasRx(STING) altered their activation state. LPS increased the CD86^+^ fraction to 86.8% and left only 0.336% CD206^+^ cells (Figure 4A). The single‐function formulations produced partial changes; the non‐functionalized dual formulation produced a larger shift, and the D4F‐functionalized dual formulation reduced CD86 positivity to 31.8% while increasing CD206 positivity to 25.5% (Figure 4A).

Immunoblotting corroborated the flow‐cytometric results. CD86 decreased, and CD206 increased across the active treatment groups, with the lowest CD86/ACTIN and highest CD206/ACTIN ratios after D4F‐CeO_2_@LNP‐CasRx(STING) treatment (Figure 4B–D). These marker changes support a shift toward a less inflammatory and more pro‐resolving macrophage state.

We next asked whether these marker changes were accompanied by functional alteration of the macrophage secretome. The cytokine heatmap revealed a broad contraction of inflammatory cytokines and chemokines together with relative enrichment of repair‐associated factors in the treatment groups, with the strongest shift again observed after D4F‐CeO_2_@LNP‐CasRx(STING) exposure (Figure 4E). This trend was substantiated by quantification of representative soluble mediators. IL‐10 increased stepwise across the treatment series, whereas CCL5, IL‐6, TGF‐β, and TNF‐α declined, with the optimized dual‐delivery group showing the largest deviation from the LPS condition (Figure 4F–J). The combined marker and secretory changes support a less inflammatory, more pro‐resolving macrophage state.

### 2.4 Reprogrammed Macrophage Secretomes Alleviate Hypoxia‐Induced Tubular Epithelial Injury

**FIGURE 5:**
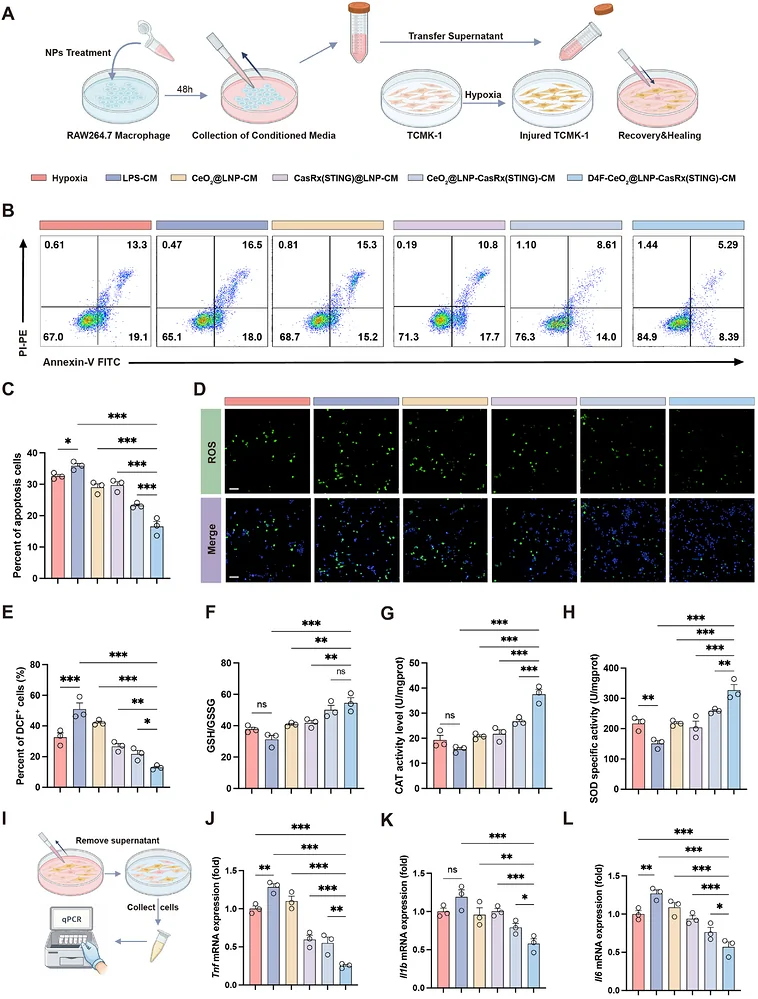
Macrophage‐conditioned media modulate hypoxia‐induced tubular injury. (A) Conditioned‐medium transfer workflow. The six recipient‐cell groups were hypoxia alone with matched fresh medium; hypoxia + LPS‐macrophage CM; and hypoxia + CM from macrophages treated with CeO_2_@LNP, CasRx(STING)@LNP, CeO_2_@LNP‐CasRx(STING), or D4F‐CeO_2_@LNP‐CasRx(STING), as labeled in the figure. (B,C) Annexin V/PI profiles and apoptosis (*n* = 3). (D,E) DCF fluorescence and ROS‐positive cells (*n* = 3). (F–H) GSH/GSSG, CAT, and SOD (*n* = 3). (I) RT–qPCR workflow. (J–L) *Tnf*, *Il1b*, and *Il6* expression (*n* = 3). Data are mean ± SD; one‐way ANOVA; ns, not significant; ^*^ *p* < 0.05, ^**^ *p* < 0.01, and ^***^ *p* < 0.001; Scale bar in D, 50 µm.

We next investigated how macrophage‐derived soluble factors influenced hypoxia‐induced tubular epithelial injury. Conditioned media were collected from RAW264.7 macrophages under the indicated treatment conditions and transferred to hypoxia‐injured TCMK‐1 cells. The six recipient groups comprised hypoxia with matched fresh medium, hypoxia with conditioned medium from LPS‐stimulated macrophages, and hypoxia with conditioned media from macrophages treated with CeO_2_@LNP, CasRx(STING)@LNP, CeO_2_@LNP‐CasRx(STING), or D4F‐CeO_2_@LNP‐CasRx(STING) (Figure 5A). Conditioned medium from LPS‐stimulated macrophages further increased apoptosis relative to the hypoxia‐only group. In contrast, conditioned media from formulation‐treated macrophages reduced this additional injury to different extents, with the lowest apoptotic fraction observed after transfer of D4F‐CeO_2_@LNP‐CasRx(STING)‐conditioned medium (Figure 5B,C).

Inflammatory macrophage‐conditioned medium also increased DCF fluorescence and reduced antioxidant capacity relative to hypoxia alone. Conditioned medium from D4F‐CeO_2_@LNP‐CasRx(STING)‐treated macrophages reduced the DCF‐positive fraction and increased the GSH/GSSG ratio, catalase activity, and superoxide dismutase activity compared with LPS‐conditioned medium (Figure 5D–H). Because the recipient TCMK‐1 cells were not directly exposed to nanoparticles in this experiment, these results reflect modification of the macrophage‐conditioned environment.

After conditioned‐medium removal, TCMK‐1 cells were analyzed by RT–qPCR (Figure 5I). LPS‐conditioned medium increased Tnf and Il6 expression beyond the hypoxia‐only group and increased Il1b relative to the treated conditioned‐medium groups. Conditioned medium from D4F‐CeO_2_@LNP‐CasRx(STING)‐treated macrophages reduced Tnf, Il1b, and Il6 expression compared with LPS‐conditioned medium (Figure 5J–L). These data are consistent with reduced apoptotic, oxidative, and inflammatory stress in the recipient tubular cells.

### 2.5 D4F‐CeO_2_@LNP‐CasRx(STING) Enhances Intratissue Accumulation in Injured Kidney Organoids and Promotes Repair in PBMC‐Coupled Organoid Cultures

**FIGURE 6:**
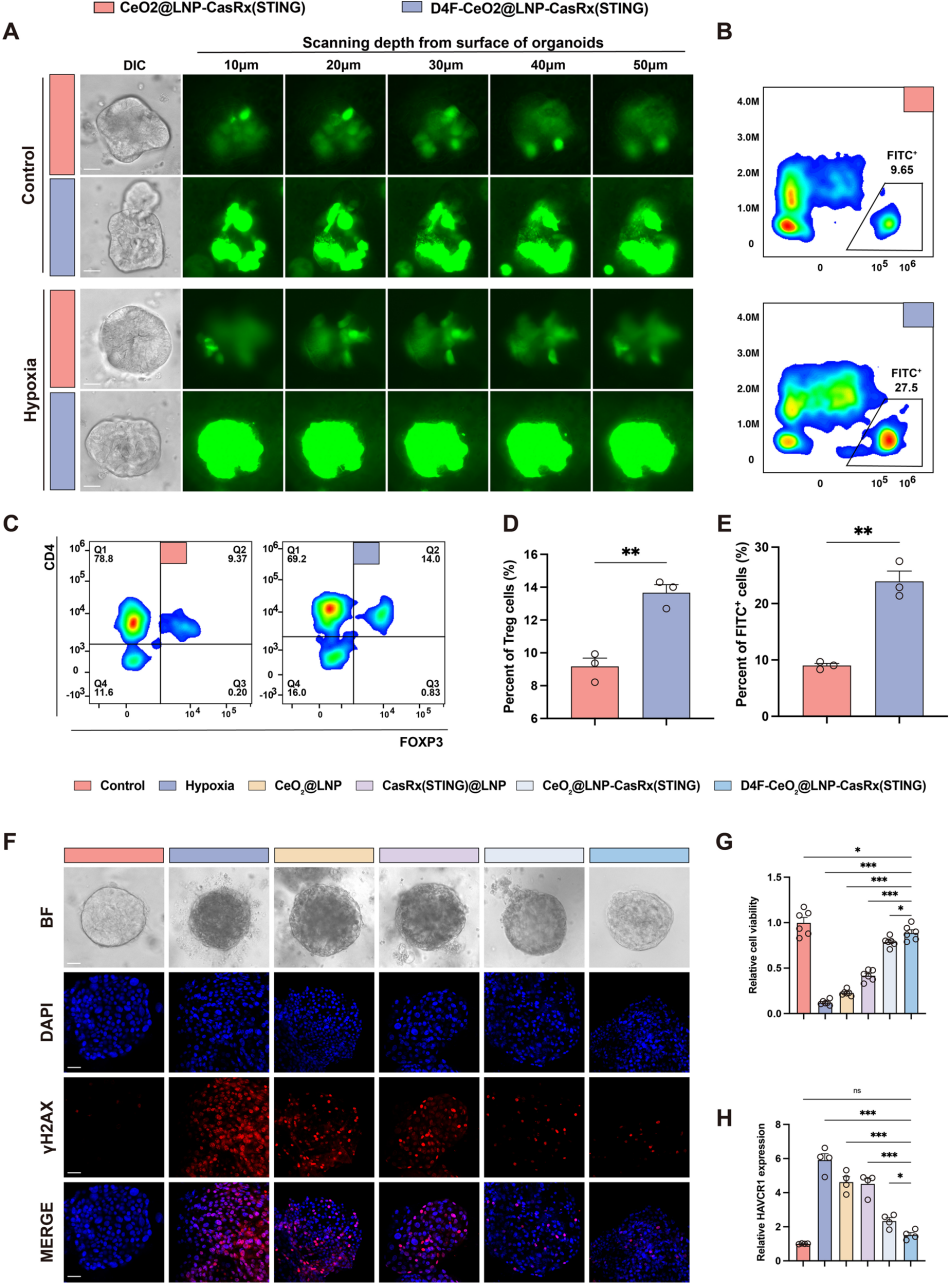
D4F functionalization improves organoid‐associated accumulation and attenuates hypoxic injury. (A) Bright‐field and fluorescence images at 10–50 µm depth under normoxia and hypoxia (*n* = 3; Scale bar, 50 µm). (B,E) Nanoparticle‐positive organoid cells and quantification. (C,D) CD4/FOXP3 flow cytometry and CD4^+^FOXP3^+^ T‐cell frequency; the complete gating sequence is provided in Figure S11. (F) DAPI/γH2AX imaging (*n* = 6; Scale bar,50 µm). (G) Relative organoid survival. (H) Relative HAVCR1 expression. Data are mean ± SD. ^*^ *p* < 0.05, ^**^ *p* < 0.01, and ^***^ *p* < 0.001.

To determine whether the nanoplatform retained these advantages in a higher‐order tissue context, we examined its distribution in PBMC‐coupled kidney organoids (workflow shown in Figure S8). Optical sectioning at depths of 10–50 µm below the organoid surface revealed stronger fluorescence from D4F‐CeO_2_@LNP‐CasRx(STING) than from the non‐functionalized formulation under both normoxic and hypoxic conditions (Figure 6A). Flow cytometry confirmed this advantage under hypoxia, with D4F functionalization increasing the FITC‐positive fraction from 9.65% to 27.5% (Figure 6B,E). The complete flow‐cytometry gating strategy is provided in Figure S9. Under normoxia, D4F functionalization likewise increased the FITC‐positive fraction, although overall positivity remained lower than under hypoxia (Figure S10). The greater FITC positivity and deeper intratissue fluorescence under hypoxia likely reflected injury‐associated macrophage recruitment and infiltration. Increased macrophage entry into the injured organoids, including deeper regions, may therefore favor macrophage‐associated accumulation of the nanoplatform.

In parallel, enhanced intratissue accumulation was also accompanied by an increased proportion of regulatory T cells in PBMC‐coupled organoids. This population was identified using the singlet→CD45→CD3→CD25→CD4/FOXP3 gating strategy shown in Figure S11. Compared with CeO_2_@LNP‐CasRx(STING), D4F‐CeO_2_@LNP‐CasRx(STING) increased the frequency of CD4^+^FOXP3^+^ T cells from approximately 9% to 14% (Figure 6C,D).

We then assessed organoid injury under hypoxia using viability, γH2AX immunofluorescence, and HAVCR1 expression as complementary readouts. Hypoxia markedly reduced organoid viability and increased γH2AX immunofluorescence and HAVCR1 expression, whereas the active formulations attenuated these changes to varying degrees. Among the hypoxic groups, D4F‐CeO_2_@LNP‐CasRx(STING) yielded the highest viability, the weakest γH2AX signal, and the lowest HAVCR1 expression (Figure 6F–H). These coordinated improvements support enhanced protection and recovery of injured organoids.

### 2.6 D4F Functionalization Enhances Renal Accumulation and Macrophage‐Associated Distribution In Vivo

**FIGURE 7:**
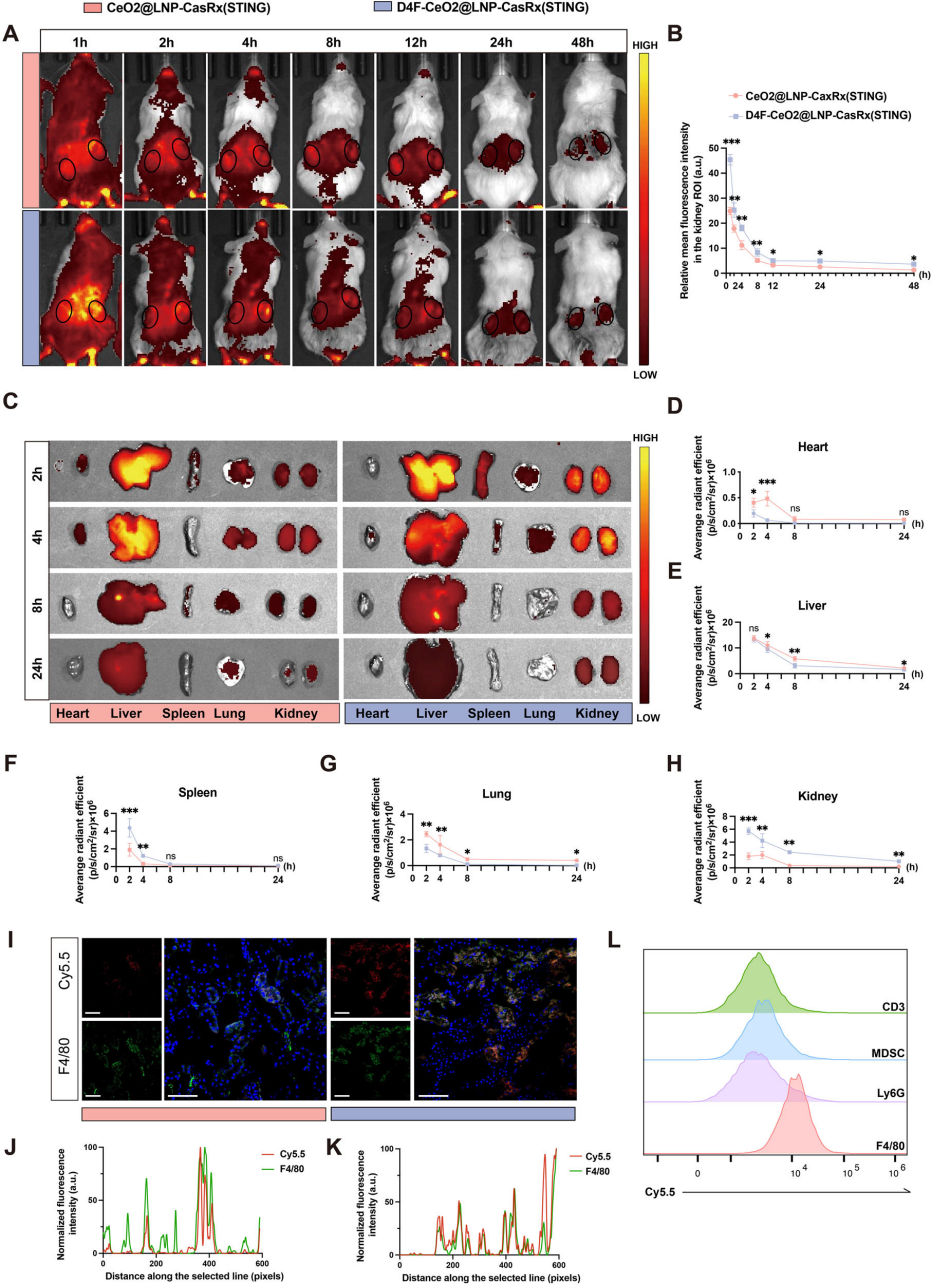
D4F functionalization alters in vivo distribution and increases macrophage‐associated renal accumulation. (A) Whole‐body Cy5.5 imaging at 1–48 h. (B) Kidney‐region fluorescence quantification. (C) Ex vivo imaging of heart, liver, spleen, lung, and kidney. (D–H) Organ‐specific radiant‐efficiency time courses. (I) Kidney Cy5.5 and F4/80 immunofluorescence (Scale bar,50 µm). (J,K) Line‐scan profiles across the selected regions. (L) Cy5.5 signal in gated peripheral CD3^+^, MDSC, Ly6G^+^, and F4/80^+^ populations. Data are mean ± SD (*n* = 3 biological replicates). Panels B and D–H were analyzed using two‐way ANOVA, with Šídák's multiple‐comparisons test for between‐group comparisons at each time point; ns, not significant; ^*^ *p* < 0.05, ^**^ *p* < 0.01, and ^***^ *p* < 0.001.

We next examined how D4F surface functionalization affected nanoparticle distribution in vivo. Serial whole‐body imaging revealed distinct signal profiles from 1 to 48 h, with stronger and more persistent kidney‐region fluorescence for D4F‐CeO_2_@LNP‐CasRx(STING) (Figure 7A). Quantification of the renal region confirmed higher fluorescence throughout the observation period (Figure 7B). Ex vivo imaging and organ‐specific time courses further revealed time‐ and organ‐dependent distribution, with the most sustained enhancement occurring in the kidney (Figure 7C–H). D4F functionalization therefore produced a biodistribution profile characterized by greater and more persistent renal accumulation.

Kidney sections were co‐stained for Cy5.5 and F4/80 to examine the cellular distribution of the renal signal. D4F functionalization resulted in greater spatial overlap between nanoparticle‐associated Cy5.5 fluorescence and F4/80^+^ macrophages (Figure 7I). Line‐scan profiles across selected regions showed closely aligned Cy5.5 and F4/80 peaks (Figure 7J,K). Peripheral blood analysis provided complementary evidence. Among the analyzed CD3^+^, MDSC, Ly6G^+^, and F4/80^+^ populations, F4/80^+^ cells showed the strongest Cy5.5 signal (Figure 7L). The agreement between renal colocalization and circulating‐cell profiles linked enhanced kidney accumulation to a macrophage‐associated distribution pattern.

Given this favorable renal distribution, we next evaluated in vivo tolerability after two closely spaced administrations. Healthy mice received two intravenous doses of D4F‐CeO_2_@LNP‐CasRx(STING) 3 h apart according to the simulated I/R dosing schedule. Histological examination at serial time points through day 45 showed preserved tissue architecture across the examined major organs (Figure S12). At day 45, RBC, HGB (g dL^−^^1^), WBC, PLT, ALT, and creatinine remained comparable to those in saline‐treated mice (Figure S13). The consistent histological, hematological, and biochemical findings support a favorable in vivo tolerability profile under the tested dosing regimen.

### 2.7 D4F‐CeO_2_@LNP‐CasRx(STING) Protects Against I/R‐Induced AKI and Attenuates Macrophage STING Signaling

**FIGURE 8:**
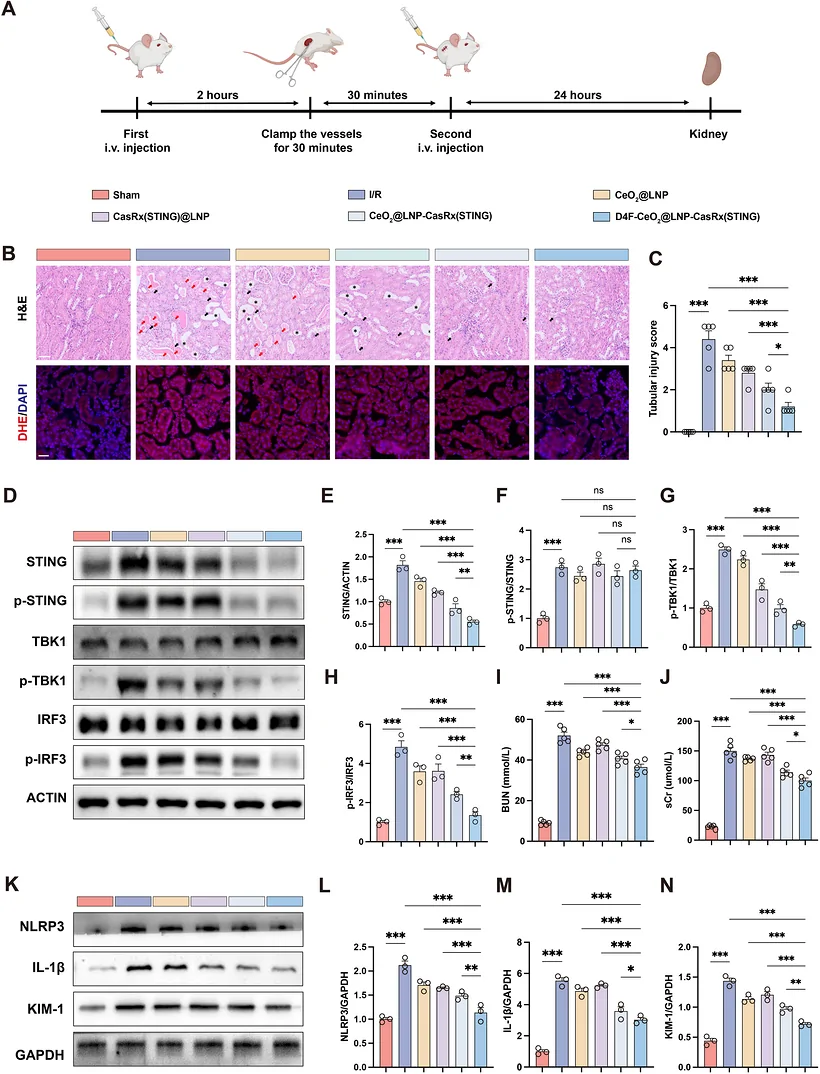
D4F‐CeO_2_@LNP‐CasRx(STING) attenuates renal I/R injury. (A) Peri‐ischemic dosing schedule. (B) H&E and DHE staining. Black arrows indicate tubular epithelial degeneration/disorganization, red arrows indicate casts, and asterisks indicate tubular dilation. Scale bars, 50 µm. (C) Blinded tubular‐injury score (*n* = 5 mice per group). (D) STING‐pathway immunoblots in sorted CD11b^+^ renal macrophages. (E–H) STING/ACTIN, p‐STING/STING, p‐TBK1/TBK1, and p‐IRF3/IRF3 (*n* = 3). (I,J) BUN and serum creatinine (*n* = 5). (K) Whole‐kidney NLRP3, IL‐1β, KIM‐1, and GAPDH immunoblots. (L–N) Corresponding ratios (*n* = 3). Data are mean ± SD; one‐way ANOVA with Tukey's multiple‐comparisons test; ns, not significant; ^*^ *p* < 0.05, ^**^ *p* < 0.01, and ^***^ *p* < 0.001.

We next evaluated therapeutic efficacy in a murine renal I/R model. The indicated formulations were administered intravenously 2 h before vascular clamping and again 30 min after restoration of renal blood flow. Kidneys were collected 24 h later (Figure 8A). I/R produced marked tubular dilation, epithelial degeneration, structural disorganization, and intratubular casts. The treatment groups showed graded preservation of tubular architecture, with the most pronounced improvement after D4F‐CeO_2_@LNP‐CasRx(STING) treatment (Figure 8B). Blinded semiquantitative scoring confirmed the lowest tubular‐injury score in this group (Figure 8C). DHE fluorescence was intense and widespread after I/R, consistent with a marked increase in intrarenal oxidative stress. Treatment reduced both the intensity and extent of the DHE signal, with the weakest fluorescence observed after D4F‐CeO_2_@LNP‐CasRx(STING) treatment (Figure 8B).

Improved tubular morphology was accompanied by recovery of renal function. I/R markedly increased blood urea nitrogen and serum creatinine. CeO_2_@LNP and CasRx(STING)@LNP each produced partial decreases, whereas the non‐functionalized dual formulation produced a greater improvement. D4F‐CeO_2_@LNP‐CasRx(STING) yielded the lowest values among the treatment groups (Figure 8I,J). PAS staining further supported preservation of tubular morphology during the acute phase (Figure S14). At two months, representative Masson‐stained sections showed less interstitial collagen deposition in the active treatment groups, consistent with sustained structural protection (Figure S15).

Because macrophage STING was a central therapeutic target, renal CD11b^+^ macrophages were isolated for pathway analysis. I/R increased STING abundance and the p‐TBK1/TBK1 and p‐IRF3/IRF3 ratios, whereas total TBK1 and IRF3 remained broadly stable. Treatment produced graded reductions in STING/ACTIN and downstream phosphorylation, with the largest decreases after D4F‐CeO_2_@LNP‐CasRx(STING) treatment (Figure 8D,E,G,H). The p‐STING/STING ratio remained comparable across the I/R and treatment groups (Figure 8F). In whole‐kidney lysates, I/R also increased NLRP3, IL‐1β, and KIM‐1. All treatment groups reduced these proteins, with the lowest levels observed in the D4F‐functionalized group (Figure 8K–N). These whole‐kidney changes paralleled the macrophage signaling response and the improvements in tubular structure and renal function.

Ex vivo culture provided a functional readout of the renal macrophage response. Under matched conditions, CD11b^+^ renal macrophages from treated I/R mice secreted less TNF‐α and IL‐6 and more IL‐10 than cells from untreated I/R mice (Figure S16). This cytokine profile was consistent with a less inflammatory, more pro‐resolving macrophage state.

### 2.8 Cell‐Resolved Spatial Transcriptomics Reveals Remodeling of the Renal Immune Niche from Inflammatory Injury Toward Repair

**FIGURE 9:**
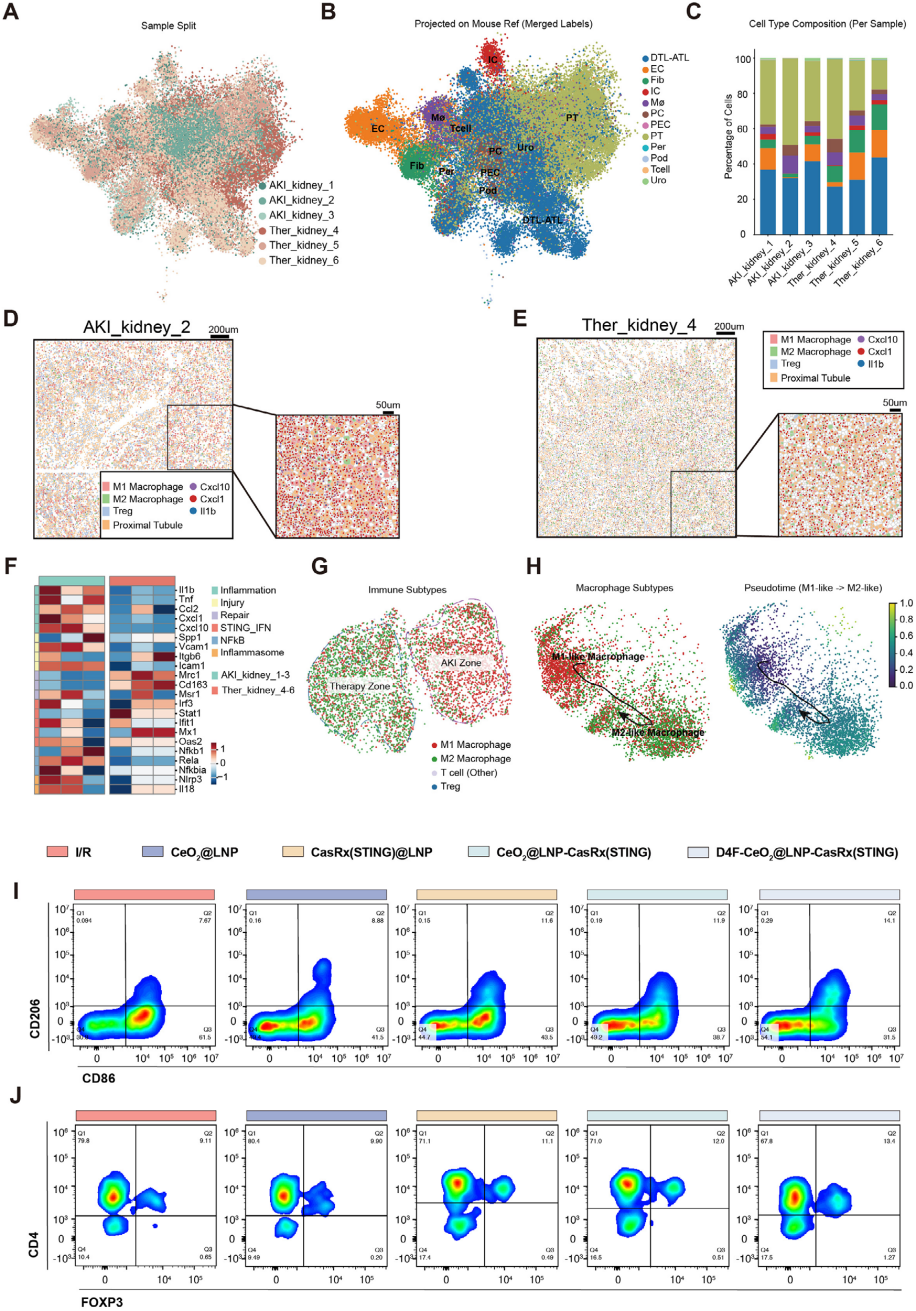
Cell‐resolved spatial transcriptomics and renal immune‐cell analyses. (A) Integrated sample distribution for three AKI and three therapy kidneys. (B) Reference‐atlas projection. (C) Cell‐type composition. (D,E) Representative AKI and therapy spatial maps. (F) Injury‐, inflammation‐, repair‐, STING/IFN‐, NF‐κB‐, and inflammasome‐related genes. (G) Immune‐subtype zoning. (H) Macrophage spatial distribution and inferred macrophage‐state continuum. (I) Renal macrophage CD86/CD206 flow cytometry. (J) Renal CD4/FOXP3 flow cytometry.

To define how the optimized therapy remodels the injured kidney in situ, we performed cell‐resolved spatial transcriptomics on six kidneys, including three AKI samples and three therapy samples. Dimensionality reduction showed that all six specimens were well represented in the integrated dataset, with each sample contributing broadly to the reconstructed spatial atlas rather than dominating a single isolated cluster (Figure 9A). Projection onto a mouse kidney reference further resolved the major epithelial, stromal, vascular, and immune compartments, including proximal tubules, distal tubule/ascending limb segments, endothelial cells, fibroblasts, podocytes, macrophages, and T‐cell populations (Figure 9B). Consistent with this annotation, the cell‐type composition plot revealed clear differences between AKI and therapy kidneys at the whole‐sample level, indicating that treatment was associated with a remodeled intrarenal cellular landscape rather than a change restricted to a single compartment (Figure 9C). Spatial maps from all six sections further supported the reproducibility of this organization across biological replicates (Figure S17).

We next examined how these transcriptomic changes were distributed within the renal tissue space. In the representative AKI section, proximal tubular regions were closely associated with inflammatory macrophage‐rich areas, and the enlarged field highlighted a local niche dominated by proximal tubules together with inflammatory/M1‐like‐enriched macrophage states and only limited pro‐resolving immune populations (Figure 9D). In contrast, the representative therapy section displayed a different architecture, in which proximal tubules were embedded within a more balanced immune microenvironment containing inflammatory/M1‐like‐enriched and pro‐resolving/M2‐like‐enriched macrophage states together with detectable Treg signals (Figure 9E). Sample‐level immune‐composition analysis further supported this pattern, showing that therapy kidneys were less dominated by the inflammatory niche seen in AKI kidneys (Figure S18). Marker‐expression dot plots were consistent with these annotations, with inflammatory/M1‐like‐enriched macrophages expressing *Cd80*, *Il1b*, *Ccl2*, and *Cxcl10*, pro‐resolving/M2‐like‐enriched macrophages expressing *Mrc1* and *Fcrls*, and Tregs expressing *Foxp3* and *Ctla4* (Figure S19).

To examine pathway‐level remodeling in space, we next compared gene programs related to injury and repair. The heatmap in Figure 9F showed that AKI kidneys were characterized by stronger expression of inflammation‐ and injury‐associated genes, including *Il1b*, *Tnf*, *Ccl2*, *Cxcl1*, *Cxcl10*, *Spp1*, *Vcam1*, Itgb6, and *Icam1*, together with enrichment of STING/IFN‐, NF‐κB‐, and inflammasome‐related genes such as *Irf3*, *Stat1*, *Ifit1*, Mx1, *Oas2*, *Nfkb1*, *Rela*, *Nlrp3*, and *Il18*. By contrast, the therapy group showed relative enrichment of repair‐associated macrophage markers including *Mrc1*, *Cd163*, and *Msr1* (Figure 9F). Immune‐subtype mapping separated the kidneys into an AKI zone enriched for inflammatory/M1‐like macrophage states and a therapy zone enriched for pro‐resolving/M2‐like macrophage states and Tregs (Figure 9G), consistent with treatment‐associated remodeling of the spatial renal immune niche.

Within the macrophage compartment, spatial transcriptomics resolved inflammatory/M1‐like‐enriched and pro‐resolving/M2‐like‐enriched states. Diffusion‐map pseudotime placed these states along an inferred continuum from an inflammatory/M1‐like‐enriched state toward a pro‐resolving/M2‐like‐enriched state (Figure 9H). Along this inferred continuum, pro‐inflammatory/M1‐like and apoptosis‐associated signatures declined, whereas anti‐inflammatory/M2‐like and tissue repair/regeneration programs increased (Figure S20). On‐site immune‐composition analysis showed the same directional association, with fewer inflammatory/M1‐like macrophages and more pro‐resolving/M2‐like macrophages in therapy sections, while other T‐cell populations changed less prominently (Figure S21). Representative flow cytometry plots from kidneys were concordant with the spatial data, showing a progressive reduction in CD86‐high/CD206‐low inflammatory macrophages and an increase in CD4^+^FOXP3^+^ T‐cell frequency after treatment, with the most evident shift in the D4F‐CeO_2_@LNP‐CasRx(STING) group (Figure 9I,J; gating strategies in Figures S22 and S23). Finally, ligand–receptor analysis predicted weaker injurious interactions involving inflammatory macrophages, proximal‐tubule injury, and immune recruitment, together with stronger pro‐resolving macrophage/Treg‐associated repair interactions (Figure S24). These data are consistent with remodeling of the renal immune niche from an inflammatory injury‐dominant state toward a repair‐permissive spatial environment.

### 2.9 Proteomic Profiling Corroborates Suppression of Injury‐Associated Inflammatory Programs and Restoration of Reparative Metabolism

To obtain an orthogonal systems‐level view of treatment response, we next compared the kidney proteomes of AKI and therapy groups. Principal component analysis showed separation between the groups, consistent with broad proteome remodeling rather than a change restricted to a few isolated proteins (Figure S25). The volcano plot also showed AKI‐associated proteins, including S100a8, Lcn2, Havcr1, and Nfkb1, and therapy‐associated antioxidant and repair‐related proteins, including Arg1, Sod2, Chil3, Cat, Gpx1, and Mrc1 (Figure S26). At the proteome level, the observed pattern was consistent with a shift away from inflammatory injury and toward a more protective molecular program.

Functional enrichment analysis provided a mechanistic framework for this transition. The pathway bubble plot showed that therapy‐associated proteins were enriched in detoxification of reactive oxygen species, peroxisome metabolism, FOXO‐mediated transcription, fatty acid metabolism, glutathione metabolism, and mitochondrial biogenesis, whereas AKI‐associated proteins were linked to NF‐κB activation, TNF signaling, apoptosis, cytosolic DNA sensing, interleukin signaling, and the senescence‐associated secretory phenotype (Figure S27). The GO functional‐signature plot further refined this pattern. AKI kidneys were dominated by cytokine‐mediated signaling, inflammatory response, cellular response to reactive oxygen species, type I interferon signaling, and apoptotic pathways, whereas therapy kidneys shifted toward cellular protein metabolism, intracellular protein transport, fatty acid catabolism and oxidation, peroxisome organization, and hypoxia‐adaptive processes (Figure S28). Together, these analyses indicate that treatment suppresses inflammation‐centered injury programs while restoring antioxidant and metabolic functions required for tissue recovery.

We next asked whether these changes were organized into coherent protein networks. The protein‐interactome map revealed a connected inflammatory module centered on Ripk1, Ripk3, Ikbkb, Nfkb1, Tnfrsf1a, Stat1, and Isg15 on the AKI side, in contrast to a therapy‐associated network enriched for antioxidant and reparative proteins including Cat, Gpx4, Gpx1, Sod2, Arg1, Mrc1, and Chil3 (Figure S29). Consistent with this network‐level redistribution, pathway heatmaps showed broad attenuation of proteins belonging to the STING/DNA‐sensing module, including Oasl1, Ifit3, Ifit1, Stat1, Oas1a, Isg15, and Ifih1, together with parallel suppression of NF‐κB pathway components such as Rela, Vcam1, Icam1, Nfkb1, Myd88, Tab1, Ikbkg, Map3k7, and Ikbkb in therapy kidneys (Figure S30). These findings indicate that the proteomic response to treatment is coordinated and pathway‐directed rather than diffuse.

Representative protein‐level comparisons further illustrated this shift. Injury‐associated proteins, including Havcr1, Lcn2, and S100a9, were lower in therapy kidneys, whereas proteins associated with tubular integrity or repair‐related immune states, including Lrp2, Arg1, and Mrc1, were higher (Figure S31). These proteomic data corroborate the spatial transcriptomic findings and are consistent with suppression of AKI‐associated inflammatory and injury programs together with restoration of antioxidant defense, metabolic adaptation, and repair‐associated protein networks.

## 3 Discussion

AKI develops through self‐reinforcing injury loops rather than a single isolated lesion [1, 16]. Oxidant accumulation damages mitochondria and tubular epithelium, danger signals released from injured cells activate innate immune pathways, and inflammatory cells then feed back on the renal parenchyma. D4F‐CeO_2_@LNP‐CasRx(STING) was designed around this coupled biology. The ceria component provided catalytic redox buffering, CasRx depleted STING transcripts, and D4F functionalization enhanced access to macrophages and the injured kidney. The component‐control formulations help distinguish these contributions. CeO_2_@LNP and CasRx(STING)@LNP each produced partial effects on different arms of the injury response; their co‐delivery broadened the protective activity, and D4F functionalization further improved productive cellular uptake and renal accumulation. The response of the optimized formulation is therefore best understood as coordinated intervention in oxidative stress, innate immune signaling, and tissue delivery.

STING lies at an important intersection between mitochondrial injury and inflammatory amplification [19, 20]. Mitochondrial DNA released from damaged cells can activate cGAS–STING signaling and sustain inflammatory injury in AKI [21]. CasRx reduced STING transcript and protein abundance. The p‐STING/STING ratio remained unchanged, whereas p‐TBK1/TBK1 and p‐IRF3/IRF3 decreased while total TBK1 and IRF3 remained largely stable. This pattern indicates that STING depletion limited downstream signaling rather than causing generalized loss of downstream proteins. The matched non‐targeting guide, two independent STING‐targeting guides, and guide‐resistant rescue construct further support sequence‐specific target engagement. In parallel, ceria‐containing formulations lowered intracellular ROS. The two modules thus addressed connected drivers of the same injury cycle: oxidative danger signaling and STING‐mediated inflammatory amplification [20, 22].

Macrophages provided the functional link between molecular target engagement and tubular protection [7, 10]. In the conditioned‐medium experiment, recipient TCMK‐1 cells were not directly exposed to nanoparticles. Their lower apoptosis, ROS accumulation, and inflammatory gene expression therefore linked altered macrophage secretion to epithelial protection. Renal CD11b^+^ macrophages from treated mice retained a similar secretory profile ex vivo. CD4^+^FOXP3^+^ T‐cell frequency also increased in PBMC‐coupled organoids and treated kidneys. The data reflect a change in relative frequency, as recruitment, survival, and proliferation were not distinguished. Viewed alongside the macrophage findings, this change suggests broader rebalancing of the renal immune environment [8, 23].

The distribution data help explain how D4F contributed to this response. D4F functionalization increased macrophage uptake, enhanced intratissue accumulation in kidney organoids, and produced stronger renal fluorescence after systemic administration. Renal Cy5.5/F4/80 overlap and the strong Cy5.5 signal in peripheral F4/80^+^ cells identified macrophages as a prominent formulation‐associated population. ApoA‐I mimetic peptides can regulate monocyte/macrophage activity and pattern‐recognition responses [17, 18]. Here, D4F also enhanced access to the cellular compartment in which STING silencing and secretory modulation were intended. The deeper organoid fluorescence under hypoxia is compatible with injury‐associated macrophage recruitment and infiltration. Direct cell tracking will be required to define the relative contributions of circulating uptake, intrarenal macrophage association, and nonimmune renal deposition.

The organoid and multi‐omics analyses extended these observations from individual cells to tissue organization. PBMC‐coupled kidney organoids retain a three‐dimensional structure and partial immune context that are absent from monolayer cultures [24, 25]. Increased viability and lower γH2AX and HAVCR1 signals showed that the protective response persisted in this more complex environment. Spatial transcriptomics placed injured proximal tubular regions near inflammatory macrophage‐rich neighborhoods with stronger STING/IFN, NF‐κB, inflammasome, and injury‐associated programs. Treated kidneys showed greater representation of pro‐resolving macrophage and Treg‐associated regions, together with predicted repair‐related cellular interactions. Pseudotime reflected an ordering of macrophage activation states rather than lineage conversion. Proteomic profiling provided an independent view, showing contraction of inflammatory and DNA‐sensing networks alongside restoration of antioxidant defense, metabolic adaptation, and repair‐associated proteins. These spatial patterns are also consistent with recent kidney atlases [5, 26]. The convergence of both datasets suggests that renal protection involved coordinated remodeling of inflammatory and metabolic networks rather than changes in a few isolated markers.

Placed in the context of current AKI nanotherapy, the distinction of this platform lies in how its components were organized around the feedback structure of the disease. Ceria nanozymes, kidney‐targeted carriers, and anti‐inflammatory nanomedicines have each shown benefit in experimental AKI [11, 13]. Here, catalytic ROS control was combined with programmable RNA‐level suppression of an inducible innate immune hub, while D4F functionalization enhanced access to a disease‐relevant phagocyte compartment. CasRx offers transcript‐level regulation without permanent genome editing [15], allowing the inflammatory component of the platform to remain sequence‐directed and potentially adaptable. The resulting design connects molecular target engagement with macrophage–tubule communication, immune composition, and spatial niche remodeling. Its modularity may allow other inducible transcripts or targeting ligands to be evaluated in future applications, although the present evidence specifically supports the STING‐directed configuration in renal I/R injury.

The treatment window remains an important consideration for translation. The efficacy study used a peri‐ischemic regimen consisting of one dose before vascular clamping and a second dose during early reperfusion. Such timing may be applicable to predictable ischemic injury during transplantation or planned surgery, whereas treatment of established AKI will require efficacy after delayed administration. Dose–response relationships, pharmacokinetics, long‐term cerium clearance, plasmid persistence, and quantitative outcomes across the AKI‐to‐CKD transition also need to be defined. The serial histology and 45‐day hematological and biochemical findings provide initial evidence of safety under the tested regimen. Dedicated studies of immunogenicity and antimicrobial host defense will nevertheless be important because the platform is designed to modify macrophage activity.

Further mechanistic resolution will require direct tracking of formulation‐bearing cells and cell‐type‐specific interrogation of STING. The relative contributions of macrophage STING, tubular STING, and other immune‐cell compartments cannot be separated by whole‐tissue analyses alone. Kidney organoids also retain limitations in maturation and immune complexity, while pseudotime and ligand–receptor analyses generate testable models of cellular organization rather than direct evidence of state transition or signaling exchange. Conditional genetic models, lineage‐resolved tracking, and perturbation of selected intercellular interactions should help establish which elements of the proposed mechanism are required for renal protection. Even with these questions open, the agreement among guide‐specific rescue experiments, macrophage‐conditioned‐medium transfer, organoid protection, renal I/R outcomes, and spatial and proteomic remodeling supports a coherent model: lowering oxidative pressure and STING signaling weakens the reciprocal reinforcement between inflammatory macrophages and injured tubules, creating a renal environment more permissive to resolution and repair.

## 4 Conclusions

In conclusion, D4F‐CeO_2_@LNP‐CasRx(STING) addresses AKI as a coupled redox and immune disorder by integrating catalytic ROS control, programmable STING transcript depletion, and macrophage‐associated renal delivery. Across macrophage, conditioned‐medium, kidney organoid, and renal I/R models, the formulation attenuated downstream TBK1 and IRF3 activation, shifted macrophage secretory activity toward a less inflammatory state, protected tubular tissue, and improved renal function and histology. Spatial transcriptomic and proteomic analyses further linked these effects to contraction of inflammatory injury programs and restoration of antioxidant, metabolic, and repair‐associated networks. The study therefore provides a preclinical rationale for RNA‐delivering nanozyme platforms that interrupt the reciprocal amplification of oxidative stress, innate immunity, and tubular injury in AKI. Evaluation in delayed‐treatment models, together with long‐term disposition and immunological safety studies, will be central to defining the translational potential of this strategy.

## 5 Experimental Section

### 5.1 Materials and Reagents

DOTAP, DSPE‐PEG2000, cholesterol, DSPE‐TK‐PEG2000, cerium oxide (CeO_2_), and the D4F peptide were purchased from Xi'an Ruixi Technology Co., Ltd. DCFH‐DA was purchased from Solarbio (Cat# D6470), and the Annexin V/propidium iodide apoptosis detection kit was purchased from GOONIE (Cat# 100–101). Primary antibodies used for western blotting included STING (Proteintech, Cat# 19851‐1‐AP), p‐STING (ABclonal, Cat# AP1369, 1:1000), TBK1 (Proteintech, Cat# 67211‐1‐Ig), p‐TBK1 (ABclonal, Cat# AP0847SP, 1:1000), IRF3 (Proteintech, Cat# 66670‐1‐Ig), p‐IRF3 (Proteintech, Cat# 80519‐2‐RR, 1:2000), ACTIN (Abways, Cat# AB0035), CD86 (Proteintech, Cat# 13395‐1‐AP), CD206 (Proteintech, Cat# 83485‐1‐RR), NLRP3 (Abways, Cat# CY5651), IL‐1β (Cell Signaling Technology, Cat# 12242), and KIM‐1 (Epizyme Biotech, Cat# R014423). The STING‐targeting CasRx construct was based on the plasmid map shown in Figure S1.

### 5.2 Synthesis of DSPE‐PEG2K‐D4F

DSPE‐PEG2K‐D4F was synthesized by coupling DSPE‐PEG2K‐NHS with the free N‐terminal amino group of D4F (DWFKAFYDKVAEKFKEAF). Briefly, 100 mg of DSPE‐PEG2K‐NHS was dissolved in 3 mL of anhydrous dimethylformamide, followed by the addition of D4F peptide and triethylamine at 1.1 and 3.0 molar equivalents, respectively. The mixture was stirred at room temperature for 12 h and then dialyzed against ultrapure water using 3.5‐kDa molecular‐weight‐cutoff tubing for 48 h. The retentate was collected and lyophilized to obtain DSPE‐PEG2K‐D4F. Product formation was confirmed by ^1H NMR spectroscopy (Figure S2B).

### 5.3 Construction of the STING‐Targeting CasRx Plasmid

The RNA‐targeting construct was derived from the pAAV‐U6‐DR‐gRNA‐CMV‐CasRx‐P2A‐GFP backbone shown in Figure S1. The CasRx direct‐repeat sequence used in the constructs was 5′‐AACCCCTACCAACTGGTCGGGGTTTGAAAC‐3′. The human STING‐targeting spacer was 5′‐TGTAATATGACCATGCCAGCCCA‐3′. The two mouse STING spacers were 5′‐CCAATGTAGTATGACCAGGCCAG‐3′ for CasRx(STING‐g1) and 5′‐ACCCGATTCTTGATGCCAGCACG‐3′ for CasRx(STING‐g2). The matched CasRx(NT) spacer was 5′‐GTGCATACTCGGAATACGGAACA‐3′. The wild‐type STING‐g1 target used as the reference for rescue‐plasmid design was 5′‐CTGGCCTGGTCATACTACATTGG‐3′, and the guide‐resistant STING‐g1 target used in the STING rescue construct was 5′‐CTCGCTTGGTCTTATTATATCGG‐3′. Plasmid input was balanced with the corresponding empty vector.

### 5.4 Preparation of D4F‐CeO_2_@LNP‐CasRx(STING)

D4F‐CeO_2_@LNP‐CasRx(STING) was assembled by stepwise electrostatic layering, as shown in Figure 2A. Positively charged aminated silica nanoparticles were incubated with the CasRx(STING) plasmid for 30 min at room temperature to form SiO_2_/pDNA complexes. For preparation of the lipid coating, DOTAP, cholesterol, DSPE‐PEG2000‐D4F, and DSPE‐TK‐PEG2000 were dissolved in 6 mL of chloroform at a molar ratio of 3:1:1:1. The solvent was removed by rotary evaporation to form a thin lipid film, which was subsequently hydrated with 4 mL of PBS and sonicated at 60°C for 1 h. The suspension was further subjected to probe sonication (Sonics and Materials Inc., USA) at 125 W in an ice‐water bath for 8 min using 5 s on/off cycles. The resulting liposomal formulation was centrifuged at 8000 rpm for 10 min at 4°C, and the purification step was repeated three times to remove free lipids and excess reactants. The purified liposomal formulation was then used to coat the SiO_2_/pDNA complexes, followed by adsorption of negatively charged ceria nanoparticles onto the surface.

For formulation optimization, SiO_2_ and plasmid were mixed at different mass ratios and assessed by agarose gel retardation and packaging‐efficiency analysis. A SiO_2_‐to‐plasmid mass ratio of 8:1 was selected, yielding a mean plasmid loading efficiency of 88.86%; ceria loading efficiency was 67.12%. CeO_2_@LNP, CasRx(STING)@LNP, CeO_2_@LNP‐CasRx(STING), and D4F‐CeO_2_@LNP‐CasRx(STING) controls were prepared by omitting the indicated component while otherwise following the matched assembly workflow. Fluorescent formulations used FITC‐tagged cargo or fluorophore‐labeled lipid; biodistribution formulations were labeled with Cy5.5. The final formulation contained pDNA and CeO_2_ at a mass ratio of 1:40 (w/w).

### 5.5 Physicochemical Characterization of Nanoparticles

Nanoparticle morphology was examined by transmission electron microscopy. Hydrodynamic diameter and PDI were measured by dynamic light scattering, and zeta potential was measured with a zeta‐potential analyzer. Elemental mapping was used to assess C, Ce, N, O, Si, S, and P. Nucleic‐acid packaging was evaluated by gel retardation. For stability testing, nanoparticles were incubated in PBS at 4°C, DMEM containing 10% FBS at 4°C, or DMEM containing 10% FBS at 37°C. Hydrodynamic diameter and PDI were measured on days 1, 3, 5, and 7, and zeta potential was compared between days 1 and 7. Measurements were performed on three independent formulations for the longitudinal DLS/PDI analysis and five independent samples for the day‐1/day‐7 zeta‐potential comparison.

### 5.6 Radical‐Scavenging and Oxygen‐Generation Assays

The catalytic antioxidant activity of the ceria‐containing formulations was evaluated by electron spin resonance, ABTS•^+^, and DPPH• assays. ABTS radical‐scavenging activity was measured using a Boxbio kit (Catalog No. AKAO021C), and DPPH radical‐scavenging activity was measured using a Boxbio kit (Catalog No. AKAO020C), in each case according to the manufacturer's instructions. Sample concentrations were expressed as CeO_2_ equivalents. Dose‐dependent radical elimination was assessed over the concentration range shown in Figure 2K,L. Catalase‐like activity was evaluated by incubating 10 mM H_2_O_2_ with D4F‐CeO_2_@LNP‐CasRx(STING) at CeO_2_‐equivalent concentrations of 25, 50, 100, and 200 µg mL^−^^1^ and monitoring dissolved oxygen in mg L^−^^1^ (Figure 2M). To test activity after storage, ABTS•^+^ and DPPH• scavenging were measured on days 1 and 7 at 4°C using five independent samples.

### 5.7 Cell Culture and In Vitro Treatments

RAW264.7 murine macrophages (BDBIO, Cat# C5142) and TCMK‐1 murine renal tubular epithelial cells (BDBIO, Cat# C5308) were maintained in complete medium supplemented with 10% fetal bovine serum and 1% penicillin‐streptomycin at 37°C in a humidified incubator containing 5% CO_2_. Human adult renal organoids (MAROs) were established from kidney biopsy or donor tissue specimens obtained from The Second Qilu Hospital of Shandong University. Tissue samples were minced, digested with collagenase for 45 min at 37°C, filtered through a 40 µm strainer, embedded in Matrigel (Corning) or basement membrane extract, and maintained in MAROs medium. Matched peripheral‐blood samples were collected from the corresponding patients or donors after written informed consent, and PBMCs were isolated by Ficoll density‐gradient centrifugation. All human‐sample collection procedures and PBMC‐related experiments were approved by the Scientific Research Ethics Committee of the Second Qilu Hospital of Shandong University (approval no. KYLL202603599). Written informed consent was obtained from all participants.

For inflammatory stimulation, RAW264.7 cells were treated with LPS (1 µg mL^−^^1^) for 24 h. The main formulation series comprised control or LPS, CeO_2_@LNP, CasRx(STING)@LNP, CeO_2_@LNP‐CasRx(STING), and D4F‐CeO_2_@LNP‐CasRx(STING). For cellular experiments, D4F‐CeO_2_@LNP‐CasRx(STING) was used at 100 µg mL^−^^1^ CeO_2_ equivalent and 2.5 µg mL^−^^1^ pDNA equivalent; the component‐control formulations were matched to the relevant CeO_2_ or pDNA equivalent. After 6 h, formulation‐containing medium was replaced with fresh medium, and cells were cultured for a total treatment period of 24 or 48 h before the indicated analysis. Hypoxic tubular and organoid injury was induced at 1% O_2_ for 48 h unless otherwise specified.

### 5.8 Cellular Delivery, ROS Measurement, and Western Blotting

For reporter‐delivery experiments, cells were incubated with GFP‐containing formulations and analyzed by fluorescence microscopy and flow cytometry. For ROS analysis, cells were stained with DCFH‐DA (Solarbio, Cat# D6470) and analyzed by fluorescence imaging or flow cytometry.

For western blotting, cells or sorted CD11b^+^ renal macrophages were lysed in RIPA buffer containing protease and phosphatase inhibitors. Protein concentration was determined by BCA assay, equal amounts were separated by SDS–PAGE and transferred to PVDF membranes, and membranes were incubated with the indicated primary antibodies overnight at 4°C. HRP‐conjugated secondary antibodies were applied for 1 h at room temperature, and bands were detected with ECL. Total STING was normalized to ACTIN, whereas p‐STING, p‐TBK1, and p‐IRF3 were normalized to their corresponding total proteins. Cells were transfected with the indicated CasRx guide and rescue constructs. At 24 h after transfection, LPS was added at 1 µg mL^−^^1^ for an additional 24 h. Cells were harvested at 48 h post‐transfection for RT–qPCR and western blot analysis.

### 5.9 Macrophage Polarization and Inflammatory Gene Profiling

RAW264.7 macrophages were stimulated with lipopolysaccharide (1 µg mL^−^^1^, 24 h) and then exposed to the indicated nanoparticle formulations. Macrophage activation was evaluated by flow cytometry using anti‐mouse CD86 (BioLegend, Cat# 105307) and CD206 (BioLegend, Cat# 141703), and by western blotting of the same markers. For inflammatory gene profiling, total RNA was extracted from treated macrophages and analyzed using a qPCR‐array platform to assess macrophage‐associated inflammatory and repair‐related transcripts. Representative soluble mediators shown in Figure 4F–J included IL‐10, CCL5, IL‐6, TGF‐β, and TNF‐α. In parallel, cell‐culture supernatants were collected after treatment, cleared by centrifugation, and analyzed by ELISA for selected cytokines to validate the direction of the qPCR‐array results at the secreted‐protein level.

### 5.10 Macrophage‐Conditioned Medium Transfer to Tubular Cells

To evaluate macrophage–tubule crosstalk, conditioned medium was collected from LPS‐stimulated RAW264.7 macrophages treated with or without the indicated formulations and transferred to hypoxia‐injured TCMK‐1 cells (Figure 5A). The hypoxia‐only group underwent the same hypoxia and recovery protocol but received matched fresh medium without macrophage‐conditioned medium. Apoptosis was measured using Annexin V/propidium iodide staining, intracellular ROS by DCFH‐DA, and redox status using GSH/GSSG, catalase, and superoxide dismutase assays.

For inflammatory‐gene analysis, conditioned medium was removed after treatment, and recipient TCMK‐1 cells were harvested. Total RNA was extracted using an RNA isolation kit, followed by reverse transcription and quantitative PCR to determine the relative expression of *Tnf*, *Il1b*, and *Il6*. Relative mRNA levels were normalized to *Gapdh* and calculated using the comparative Ct (2^−ΔΔCt) method. Primer sequences are provided in the Supporting Information.

### 5.11 Kidney Organoid Culture, Penetration Analysis, and PBMC Co‐Culture

According to our previously established kidney organoid culture protocol [27], adult renal organoids were maintained in MAROs medium until they reached the stage selected for the present study. For organoid‐based experiments, organoids were collected in cold Advanced DMEM/F12 medium and centrifuged at 300 × g for 5 min at 4°C to remove residual basement matrix, followed by gentle mechanical dissociation into small fragments. For penetration assays, organoid fragments were seeded onto culture wells pre‐coated with Matrigel diluted 1:8 in organoid culture medium and allowed to recover before exposure to fluorescently labeled CeO_2_@LNP‐CasRx(STING) or D4F‐CeO_2_@LNP‐CasRx(STING). Optical sectioning was subsequently performed at depths of 10, 20, 30, 40, and 50 µm from the organoid surface under normoxic or hypoxic conditions, and nanoparticle‐positive cells were quantified by flow cytometry after enzymatic dissociation into single‐cell suspensions.

For PBMC‐coupled organoid studies, PBMCs were mixed with kidney organoid fragments at an approximate 1:1 ratio and seeded onto Matrigel diluted 1:8. After the indicated treatments under normoxia or hypoxia, human CD4^+^CD25^+^FOXP3^+^ T cells were analyzed by flow cytometry, and organoid injury was assessed by bright‐field imaging, γH2AX immunofluorescence, viability, and HAVCR1/KIM‐1 readouts.

### 5.12 In Vivo Biodistribution and Murine Renal Ischemia‐Reperfusion Injury Model

All animal procedures were conducted in accordance with institutional guidelines and ARRIVE recommendations and were approved under protocol KYLL202603599 by the Scientific Research Ethics Committee of the Second Qilu Hospital of Shandong University; the same approval number covered the human kidney‐tissue/PBMC and animal experiments. Female BALB/c mice (6–8 weeks old, approximately 20 g) were used. For fluorescence biodistribution, labeled nanoparticles were administered intravenously at the same per‐administration doses specified below for the therapeutic studies (20 mg kg^−^^1^ CeO_2_ equivalent and 0.5 mg kg^−^^1^ pDNA equivalent) and imaged at 1, 2, 4, 8, 12, 24, and 48 h using an IVIS SPECTRUM in vivo imaging system. Images were analyzed using Living Image version 4.3.1. Major organs were collected for ex vivo imaging, and kidney sections were examined for Cy5.5 and F4/80.

For therapeutic studies, mice were randomly assigned to Sham, I/R, CeO_2_@LNP, CasRx(STING)@LNP, CeO_2_@LNP‐CasRx(STING), or D4F‐CeO_2_@LNP‐CasRx(STING). Mice were anesthetized with inhaled isoflurane and maintained on a temperature‐controlled heating pad. The first tail‐vein injection was given 2 h before 30 min of renal vascular clamping, and the second was given 30 min after restoration of renal blood flow; kidneys were collected 24 h later (Figure 8A). Each administration delivered 20 mg kg^−^^1^ CeO_2_ equivalent and 0.5 mg kg^−^^1^ pDNA equivalent, giving cumulative doses of 40 mg kg^−^^1^ CeO_2_ equivalent and 1.0 mg kg^−^^1^ pDNA equivalent. Component‐control formulations were matched to their corresponding CeO_2_ or pDNA content. The regimen was selected to span the peri‐ischemic and early reperfusion phases; it was not designed as a delayed‐treatment protocol.

### 5.13 Renal Function Tests, Histology, Immunofluorescence, and Flow Cytometry

Blood urea nitrogen and serum creatinine were measured using routine biochemical assays. Kidney sections were stained with H&E, PAS, or Masson's trichrome, and oxidative stress was assessed by DHE staining. Blinded semiquantitative tubular‐injury scoring was performed on 10 randomly selected, non‐overlapping fields per mouse at 200× total magnification using 8‐µm‐thick kidney sections. Tubular injury was scored according to the percentage of affected tubules: 0, none; 1, ≤10%; 2, 11%–25%; 3, 26%–50%; 4, 51%–75%; and 5, >75%. Field‐level scores were averaged to obtain one value per mouse, with the mouse treated as the experimental unit (*n* = 5 mice per group). For the longitudinal safety cohort, healthy mice were not subjected to renal pedicle clamping. They received two intravenous administrations 3 h apart at simulated time points corresponding to 2 h before clamping and 30 min after restoration of blood flow in the I/R protocol. The cumulative doses were 40 mg kg^−^^1^ CeO_2_ equivalent and 1.0 mg kg^−^^1^ pDNA equivalent. Major‐organ H&E was examined at 5, 10, 15, 30, and 45 days (*n* = 3 mice per time point; scale bar, 50 µm; Figure S12). At 45 days, RBC, HGB (g dL^−^^1^), WBC, PLT, ALT, and creatinine were measured as indicated in Figure S13.

For kidney flow cytometry, renal tissues were enzymatically dissociated into single‐cell suspensions, followed by red‐blood‐cell lysis and antibody staining. Macrophage CD86/CD206 and CD4^+^CD25^+^FOXP3^+^ T‐cell analyses used the panels listed in the Supporting Information. For Figure 8D–H, the CD11b^+^ renal macrophage fraction was isolated by flow sorting before protein extraction. For the ex vivo functional experiment, CD11b^+^ renal macrophages from untreated or D4F‐CeO_2_@LNP‐CasRx(STING)‐treated I/R mice were cultured under matched conditions, and TNF‐α, IL‐6, and IL‐10 in the supernatants were measured by ELISA. NLRP3, IL‐1β, and KIM‐1 in Figure 8K–N were measured in whole‐kidney lysates.

### 5.14 Cell‐Resolved Spatial Transcriptomic Analysis

Cell‐resolved spatial transcriptomics was performed on formalin‐fixed, paraffin‐embedded kidney sections using the CosMx SMI platform and CosMx 1K plex Mouse Universal Cell Characterization Panel. Cell segmentation used DAPI, CD298/B2M, PanCK, and CD45 staining. Three AKI and three therapy sections were processed using the manufacturer's workflow. Quality control included cell‐level filtering by transcript count, negative‐probe proportion, complexity, and area. Fields of view with fewer than 100 mean transcripts per cell were removed, and negative‐probe performance and target detection over background were assessed. Filtered AnnData objects were mapped to the Kirita 2020 mouse kidney reference atlas using Scanpy ingest and genes shared between the reference and query datasets.

Transferred labels defined the epithelial, stromal, vascular, and immune compartments and enabled spatial comparisons between AKI and therapy sections. Immune analyses included targeted visualization of inflammation, injury, repair, STING/IFN, NF‐κB, and inflammasome programs. Macrophage and T‐cell subtypes were assigned using canonical markers (Figure S19). Macrophage‐state trajectories were inferred by diffusion‐map pseudotime and oriented from inflammatory injury toward repair based on *Cxcl10*‐associated inflammatory behavior. Condition‐specific ligand–receptor scores were calculated from mean expression in relevant sender and receiver populations.

### 5.15 Proteomic Profiling and Bioinformatic Analysis

Kidney proteomic profiling was performed on frozen AKI and therapy kidney tissues using an Orbitrap Astral‐based data‐independent acquisition workflow. Kidney tissues were ground under liquid nitrogen, lysed in 8 M urea containing 1% protease inhibitor, sonicated, cleared by centrifugation at 4°C and 15 000 × g for 10 min, and quantified by BCA assay. Equal amounts of protein were processed according to the core‐facility digestion workflow with trypsin, reduction with 5 mM dithiothreitol, alkylation with 15 mM iodoacetamide, acidification with 10% trifluoroacetic acid, desalting on Strata X cartridges, and peptide quantification using the Pierce Quantitative Peptide Assays & Standards kit. Peptides were separated on a Vanquish Neo nanoLC system and analyzed on an Orbitrap Astral mass spectrometer in positive‐ion DIA mode. MS1 scans covered 380–980 m/z at a resolution of 240 000 at 200 m/z with a normalized AGC target of 500% and a maximum injection time of 5 ms. MS2 acquisition used 299 DIA windows with a 2 m/z isolation window, HCD collision energy of 25 eV, a normalized AGC target of 500%, and a maximum injection time of 3 ms. Raw DIA data were processed in DIA‐NN using trypsin specificity with one missed cleavage, Carbamidomethyl(C) as a fixed modification, Oxidation(M) and Acetyl(protein N‐terminus) as variable modifications, and protein identifications filtered at a false‐discovery rate of <1%.

Differential‐protein and pathway‐level visualizations were generated from the DIA dataset. Representative markers of injury and repair, including Havcr1, Lcn2, S100a9, Lrp2, Arg1, and Mrc1, were displayed as box plots. Gene‐set enrichment and pathway‐level comparisons were used to distinguish AKI‐associated and therapy‐associated biological programs.

### 5.16 Statistical Analysis

Statistical analyses were performed using GraphPad Prism version 11.0. Data are presented as mean ± standard deviation (SD). Normality and homogeneity of variance were assessed before parametric testing. Statistical tests were selected according to the experimental design, and the sample sizes, experimental units, and multiple‐comparison procedures are specified in the corresponding figure legends. All tests were two‐sided, and *p* < 0.05 was considered statistically significant.

## Author Contributions

Weibo Chen: Data curation, Software, Validation, Visualization, Writing – original draft. Ming Li: Conceptualization, Investigation, Visualization. Xuesheng Wang: Conceptualization, Data curation, Methodology. Jiajia Sun: Data curation, Investigation. Ziyuan Zhang: Data curation, Software. Yongfeng Gong: Conceptualization, Formal analysis, Methodology. Benkang Shi: Conceptualization, Formal analysis. Nengwang Yu: Conceptualization, Methodology. Yunuo Mao: Conceptualization, Methodology. Shengtian Zhao: Conceptualization, Resources, Supervision. Xiulin Zhang: Conceptualization, Funding acquisition, Project administration, Resources, Writing – review & editing. Liqiang Guo: Conceptualization, Funding acquisition, Resources, Supervision, Writing – review & editing.

## Ethics Statement

Human kidney tissue and matched peripheral blood were obtained after written informed consent. The human kidney‐tissue/PBMC studies and animal experiments were approved under protocol KYLL202603599 by the Scientific Research Ethics Committee of the Second Qilu Hospital of Shandong University. The same approval number covered both the human/PBMC and animal experiments.

## Conflicts of Interest

The authors declare no conflict of interest.

## Supporting information




**Supporting File**: advs77517‐sup‐0001‐SuppMat.docx.

## Data Availability

The data that support the findings of this study are available in the supplementary material of this article.
