# Supplementary material for: D4F‐Functionalized Ceria Nanozyme‐CasRx Platform Suppresses STING and Reprograms the Renal Immune Niche in Acute Kidney Injury
Source: Adv Sci (Weinh). 2026 Sep 6:e77517. Online ahead of print. doi: 10.1002/advs.77517 (PMC13547695; doi:10.1002/advs.77517)
Supplement: Supplementary file 1 [file ADVS-9999-e77517-s001.docx]

**Supporting Information**

*for*

**D4F-Functionalized Ceria Nanozyme-CasRx Platform Suppresses STING and Reprograms the Renal Immune Niche in Acute Kidney Injury**

*Weibo Chen¹˒⁴˒†, Ming Li²˒†, Xuesheng Wang³˒†, Jiajia Sun¹, Ziyuan Zhang¹, Yongfeng Gong¹, Benkang Shi¹, Nengwang Yu¹, Yunuo Mao⁵, Shengtian Zhao¹˒*, Xiulin Zhang⁴˒*, Liqiang Guo¹˒**

1 Department of Urology, Qilu Hospital, Cheeloo College of Medicine, Shandong University; Jinan, China.

2 Department of Ultrasound, Shandong Provincial Hospital Affiliated to Shandong First Medical University; Jinan, China.

3 Department of Urology, Linyi People's Hospital, Linyi, China.

4 Department of Urology, The Second Qilu Hospital of Shandong University, Cheeloo College of Medicine, Shandong University, Jinan, China.

5 Department of Systems Biomedicine, School of Basic Medical Sciences, Cheeloo College of Medicine, Shandong University, Jinan, Shandong 250012, China.

† These authors contributed equally.

**Correspondence:**

Shengtian Zhao, E-mail: zhaoshengtian@sdu.edu.cn

Xiulin Zhang, E-mail: zhangxiulin1965@email.sdu.edu.cn

Liqiang Guo, E-mail: glq_66@sdu.edu.cn

**Detailed Materials and Reagents**

- DCFH-DA was purchased from Solarbio (Cat# D6470). The Annexin V/propidium iodide apoptosis detection kit was purchased from GOONIE (Cat# 100-101).
- Primary antibodies used for western blotting were STING (Proteintech, Cat# 19851-1-AP), p-STING (ABclonal, Cat# AP1369, 1:1000), TBK1 (Proteintech, Cat# 67211-1-Ig), p-TBK1 (ABclonal, Cat# AP0847SP, 1:1000), IRF3 (Proteintech, Cat# 66670-1-Ig), p-IRF3 (Proteintech, Cat# 80519-2-RR, 1:2000), ACTIN (Abways, Cat# AB0035), CD86 (Proteintech, Cat# 13395-1-AP), CD206 (Proteintech, Cat# 83485-1-RR), NLRP3 (Abways, Cat# CY5651), IL-1β (Cell Signaling Technology, Cat# 12242), and KIM-1 (Epizyme Biotech, Cat# R014423).

**Guide Sequences and RT-qPCR Primers**

- CasRx direct-repeat sequence: 5′-AACCCCTACCAACTGGTCGGGGTTTGAAAC-3′.
- Human STING-targeting spacer: 5′-TGTAATATGACCATGCCAGCCCA-3′.
- Mouse STING-targeting spacer 1, CasRx(STING-g1): 5′-CCAATGTAGTATGACCAGGCCAG-3′. Mouse STING-targeting spacer 2, CasRx(STING-g2): 5′-ACCCGATTCTTGATGCCAGCACG-3′. Non-targeting spacer, CasRx(NT): 5′-GTGCATACTCGGAATACGGAACA-3′. Wild-type STING-g1 target used as the reference for rescue-plasmid design: 5′-CTGGCCTGGTCATACTACATTGG-3′. Guide-resistant STING-g1 target used in the STING rescue construct: 5′-CTCGCTTGGTCTTATTATATCGG-3′. Cells were transfected with the indicated CasRx guide and rescue constructs. At 24 h after transfection, LPS was added at 1 μg mL⁻¹ for an additional 24 h. Cells were harvested at 48 h post-transfection for RT–qPCR and western blot analysis.
- *Tnf* primers: forward 5′-CATCTTCTCAAAATTCGAGTGACAA-3′, reverse 5′-TGGGAGTAGACAAGGTACAACCC-3′.
- *Il1b* primers: forward 5′-GCAACTGTTCCTGAACTCAACT-3′, reverse 5′-ATCTTTTGGGGTCCGTCAACT-3′.
- *Il6* primers: forward 5′-TAGTCCTTCCTACCCCAATTTCC-3′, reverse 5′-TTGGTCCTTAGCCACTCCTTC-3′.
- *Gapdh* primers: forward 5′-AGGTCGGTGTGAACGGATTTG-3′, reverse 5′-TGTAGACCATGTAGTTGAGGTCA-3′.

**Additional Nanoparticle Formulation Details**

- For D4F-functionalized nanoparticles, DOTAP, D4F-conjugated PEG-lipid, cholesterol, and DSPE-TK-PEG2000 were combined at a molar ratio of 3:1:1:1.
- SiO2 and plasmid were optimized at a mass ratio of 8:1, giving a mean plasmid loading efficiency of 88.86%.
- The ceria loading efficiency in the final nanoplatform was 67.12%. The final formulation contained pDNA and CeO₂ at a mass ratio of 1:40 (w/w).
- Fluorescent formulations for organoid penetration were prepared using FITC-tagged cargo or fluorophore-labeled lipid components, and biodistribution formulations were labeled with Cy5.5. ABTS radical-scavenging activity was measured using a Boxbio kit (Catalog No. AKAO021C), and DPPH radical-scavenging activity was measured using a Boxbio kit (Catalog No. AKAO020C), according to the manufacturer’s instructions; sample concentrations were expressed as CeO₂ equivalents. For Figure 2, the doses in panels J and K are CeO₂-equivalent concentrations expressed in μg mL⁻¹, and panel M reports dissolved oxygen in mg L⁻¹ against CeO₂-equivalent concentration. Panels F–H, I, and L–M used n = 5, n = 3, and n = 3, respectively; no groupwise statistical comparison was performed for panels F–M.

**Cell Sources, Organoid Culture, and PBMC Information**

- RAW264.7 murine macrophages were obtained from BDBIO (Cat# C5142).
- TCMK-1 murine renal tubular epithelial cells were obtained from BDBIO (Cat# C5308).
- Human adult renal organoids (MAROs) were established from kidney biopsy or donor tissue specimens obtained from The Second Qilu Hospital of Shandong University. All human kidney-tissue collection procedures were approved under protocol KYLL202603599 by the Scientific Research Ethics Committee of the Second Qilu Hospital of Shandong University, and written informed consent was obtained from all participants before sample collection. Tissues were minced, digested with collagenase for 45 min at 37 °C, passed through a 40 μm filter, and embedded in Matrigel (Corning) or basement membrane extract (BME, R&D Systems) for organoid culture.
- The basal organoid medium consisted of Advanced DMEM/F12 supplemented with penicillin/streptomycin, HEPES, GlutaMAX, B27, N-acetylcysteine (1 mmol L−1), EGF (50 ng mL−1), Y-27632 (10 μM), A83-01 (5 μM), Primocin (0.1 mg mL−1), and recombinant human R-spondin-1 (100 ng mL−1). The MAROs medium further contained retinoic acid (2 μM), GDNF (100 ng mL−1), and FGF20 (100 ng mL−1). Additional factors used during organoid maintenance or differentiation included FGF1, FGF2, FGF7, FGF8, FGF9, and BMP7 (each at 100 ng mL−1), CHIR99021 (5 μM for maintenance and 2 μM in differentiation medium), LDN193189 (5 μM), TGF-α, ICG001 (5 μM), and IWR-1 (5 μM).
- For differentiation, MAROs medium was supplemented with 8-bromo-cAMP (100 μM), and additional differentiation-supporting factors included forskolin (10 μM), argipressin (100 nM), and aldosterone (2 μM). Once organoids reached the stage selected for experimentation, they were collected in cold Advanced DMEM/F12 medium and centrifuged at 300 × g for 5 min at 4 °C to remove residual basement matrix. The organoids were then gently mechanically dissociated into small fragments for subsequent penetration or co-culture assays.
- PBMC-related experiments were conducted under protocol KYLL202603599. Matched peripheral-blood samples were collected from the corresponding renal-tissue donors or patients after written informed consent, and PBMCs were isolated from fresh blood samples by Ficoll density-gradient centrifugation for subsequent co-culture experiments. For PBMC-coupled organoid cultures, PBMCs were prepared in advance and mixed with organoid fragments at an approximate 1:1 ratio. To facilitate cell movement and integration, Matrigel diluted 1:8 in organoid culture medium was used for pre-coating of the culture wells, after which the organoid-PBMC mixture was seeded on top of the pre-coated matrix. Organoids alone and PBMCs alone were cultured under the same conditions as control groups.
- For nanoparticle-delivery studies, organoid fragments were treated with fluorescently labeled CeO₂@LNP-CasRx(STING) or D4F-CeO₂@LNP-CasRx(STING) under normoxic or hypoxic conditions, followed by optical sectioning at defined depths from the organoid surface and flow-cytometric quantification after dissociation into single-cell suspensions. For immune-co-culture experiments, PBMC-coupled organoids were exposed to the indicated nanoparticle formulations under normoxic or hypoxic conditions, and human CD4⁺CD25⁺FOXP3⁺ T-cell frequency was analyzed by flow cytometry. Bright-field morphology, viability, γH2AX-associated DNA-damage response, and HAVCR1-related injury readouts were assessed in parallel to evaluate organoid protection.
- For immunofluorescence imaging, organoids or PBMC-coupled organoids were seeded into imaging chambers or plates pre-coated with diluted Matrigel. Samples were fixed with 4% paraformaldehyde for 10 min at room temperature, washed three times with PBS, and permeabilized with PBS containing 0.2% Triton X-100 for 5 min. After permeabilization, samples were blocked with 1% bovine serum albumin in PBS for 1 h at room temperature.
- Primary antibodies diluted in 1% bovine serum albumin were incubated with the samples overnight at 4 °C, followed by PBS washing and incubation with the corresponding secondary antibodies for 1 h at room temperature. Nuclei were counterstained with DAPI, and fluorescence images were acquired by confocal microscopy. Representative bright-field and fluorescence images were used to evaluate nanoparticle penetration, organoid morphology, and γH2AX-associated injury signals in the indicated treatment groups. Unless otherwise indicated, hypoxic injury in organoids was induced at 1% O2 for 48 h.

**Flow-Cytometry Antibody Panels**

- Mouse panel: CD86 (BioLegend, Cat# 105307), CD206 (BioLegend, Cat# 141703), CD45 (Invitrogen, Cat# 48-0451-80), CD3e (BioGems, Cat# 05122-70-25), CD4 (BioLegend, Cat# 100405), CD25 (BioLegend, Cat# 102007), and FOXP3 (BD, Cat# 560401).
- Human panel: CD11b (BioLegend, Cat# 393113), CD86 (BioLegend, Cat# 374214), CD206 (BioLegend, Cat# 321104), CD3 (BioLegend, Cat# 317335), CD4 (BioLegend, Cat# 317407), CD25 (BioLegend, Cat# 302605), and FOXP3 (BioLegend, Cat# 320113). The Figure S11 T-cell gating sequence is singlet→CD45→CD3→CD25→CD4/FOXP3.

**Additional Animal Study Information**

- Female BALB/c mice (6-8 weeks old, approximately 20 g) were used for in vivo experiments.
- Mice were anesthetized with inhaled isoflurane and maintained on a temperature-controlled heating pad throughout the procedure. Perioperative monitoring, analgesia, and supportive care were provided as needed to minimize pain and distress.
- All human kidney-tissue/PBMC and animal procedures were approved under protocol KYLL202603599 by the Scientific Research Ethics Committee of the Second Qilu Hospital of Shandong University. The same approval number covered both human/PBMC and animal experiments. Serial in vivo and ex vivo fluorescence imaging was performed using an IVIS SPECTRUM in vivo imaging system, and images were analyzed using Living Image version 4.3.1. Figure 7 represents n = 3 biological replicates; panels B and D–H were analyzed using two-way repeated-measures ANOVA with Šídák’s multiple-comparisons test for between-group comparisons at each time point.

Cell-Resolved Spatial Transcriptomic and Proteomic Workflow

- Cell-resolved spatial transcriptomic profiling was performed on formalin-fixed paraffin-embedded kidney sections using the CosMx SMI platform with the CosMx 1K plex Mouse Universal Cell Characterization Panel. Cell segmentation was carried out using DAPI, CD298/B2M, PanCK, and CD45. The six kidney sections analyzed in this study included AKI_kidney_1–3 and Ther_kidney_4–6, comprising 48 fields of view in total. According to the platform quality-control report, cell filtering considered minimum counts, the proportion of negative probes, transcriptomic complexity, and cell area; low-quality FOVs were flagged when the mean transcript count per cell was <100; additional negative-probe QC and target-detection-over-background assessments were applied before downstream analysis. Filtered spatial-expression matrices and coordinates were exported from the manufacturer workflow and analyzed as AnnData objects in Python, followed by mapping to the Kirita 2020 mouse kidney reference atlas using the Scanpy ingest workflow on shared genes. Macrophage pseudotime was interpreted as an inferred state continuum and not as evidence of lineage conversion; ligand–receptor scores were interpreted as predicted interactions.
- For proteomic profiling, frozen mouse kidney tissues were pulverized under liquid nitrogen and lysed in 8 M urea containing 1% protease inhibitor, followed by ultrasonic disruption, centrifugation at 4 °C and 15,000 g for 10 min, and protein quantification by BCA assay. Samples were processed according to the core-facility tissue workflow with trypsin digestion, reduction using 5 mM dithiothreitol at 56 °C for 30 min, alkylation using 15 mM iodoacetamide at room temperature in the dark for 15 min, acidification with 10% trifluoroacetic acid to pH 2–3, desalting on Strata X cartridges, vacuum drying, and peptide quantification using the Pierce Quantitative Peptide Assays & Standards kit.

Peptides were separated on a Vanquish Neo nano-flow LC system and analyzed on an Orbitrap Astral mass spectrometer in positive-ion DIA mode. MS1 acquisition covered 380-980 m/z at 240,000 resolution (at 200 m/z) with a normalized AGC target of 500% and a maximum injection time of 5 ms. MS2 acquisition used 299 DIA windows with a 2 m/z isolation window, HCD collision energy of 25 eV, a normalized AGC target of 500%, and a maximum injection time of 3 ms. Raw DIA files were processed in DIA-NN using trypsin specificity with one missed cleavage, Carbamidomethyl(C) as a fixed modification, Oxidation(M) and Acetyl(protein N-terminus) as variable modifications, and protein identifications filtered at FDR <1%.

Statistical analyses were performed using GraphPad Prism version 11.0.0. Blinded semiquantitative tubular-injury scoring was performed on 10 randomly selected, non-overlapping fields per mouse at 200× total magnification using 8-μm-thick kidney sections. Tubular injury was scored according to the percentage of affected tubules: 0, none; 1, ≤10%; 2, 11%–25%; 3, 26%–50%; 4, 51%–75%; and 5, >75%. Field-level scores were averaged to obtain one value per mouse, with the mouse treated as the experimental unit (n = 5 mice per group).

Abbreviations

| **Abbreviation** | **Definition** |
| --- | --- |
| AKI | acute kidney injury |
| ALT | alanine aminotransferase |
| BUN | blood urea nitrogen |
| CAT | catalase |
| CeO₂ | cerium dioxide |
| CM | conditioned medium |
| D4F | D-4F, an 18-residue apolipoprotein A-I mimetic peptide composed of D-amino acids; Ac-DWFKAFYDKVAEKFKEAF-NH₂ |
| DCF | 2′,7′-dichlorofluorescein |
| DHE | dihydroethidium |
| DLS | dynamic light scattering |
| DPPH | 2,2-diphenyl-1-picrylhydrazyl |
| ESR | electron spin resonance |
| FBS | fetal bovine serum |
| H&E | hematoxylin and eosin |
| HGB | hemoglobin |
| I/R; IRI | ischemia–reperfusion; ischemia–reperfusion injury |
| LNP | lipid nanoparticle |
| PBMC | peripheral blood mononuclear cell |
| PDI | polydispersity index |
| PLT | platelet count |
| RBC | red blood cell count |
| ROS | reactive oxygen species |
| RT–qPCR | reverse-transcription quantitative polymerase chain reaction |
| STING | stimulator of interferon genes |
| Treg | regulatory T cell |
| WBC | white blood cell count |

**Figure S1.** Plasmid map of the pAAV-U6-DR-gRNA-CMV-CasRx-P2A-GFP construct used for CasRx-mediated STING silencing.

**Figure S2.** Synthesis and structural verification of DSPE-PEG2K-D4F. (A) Synthetic scheme. (B) ¹H NMR spectrum confirming conjugation of D4F.

**Figure S3.** CeO₂ standard curve determined by atomic absorption spectroscopy.

**Figure S4**. Physicochemical and antioxidant stability of D4F-CeO₂@LNP-CasRx(STING). (A) Polydispersity index (PDI) of D4F-CeO₂@LNP-CasRx(STING) during incubation in PBS at 4 °C, DMEM supplemented with 10% FBS at 4 °C, or DMEM supplemented with 10% FBS at 37 °C for 1, 3, 5, and 7 days (n = 3 independent formulations). (B–D) Zeta potential, ABTS•⁺ elimination, and DPPH• elimination, respectively, on days 1 and 7 (n = 5 independent samples). Data are presented as mean ± SD. Day 1 and day 7 values in panels B–D were compared using two-tailed paired Student’s t-tests. ns, not significant.

**Figure S5.** CCK-8 analysis of RAW264.7 viability after exposure to ceria nanoparticles at the indicated concentrations. Data are mean ± SD (n = 5 per group); **P* < 0.05, ***P* < 0.01, and ****P* < 0.001.

**Figure S6**. Hemolysis assay of the indicated nanoparticle formulations with positive and negative controls.

**Figure S7.** Validation of CasRx-mediated STING silencing with two independent STING-targeting guides and a guide-resistant rescue construct. Cells were transfected with the indicated CasRx guide and rescue constructs. At 24 h after transfection, LPS was added at 1 μg mL⁻¹ for an additional 24 h. Cells were harvested at 48 h post-transfection for RT–qPCR and western blot analysis. (A) STING mRNA after PBS, empty vector, CasRx(NT), CasRx(STING-g1), CasRx(STING-g2), or CasRx(STING-g1) + guide-resistant STING (n = 5 biological replicates). (B) Immunoblots. (C–F) STING/ACTIN, p-STING/STING, p-TBK1/TBK1, and p-IRF3/IRF3 (n = 3). Data are mean ± SD; one-way ANOVA with Šídák’s multiple-comparisons test; ns, not significant; **P* < 0.05, ***P* < 0.01, and ****P* < 0.001 versus PBS; *^#^P* < 0.05 and *^##^P* < 0.01 versus CasRx(STING-g1).

**Figure S8.** PBMC-coupled kidney organoid workflow under normoxic and hypoxic conditions.

**Figure S9.** Representative complete gating sequence for nanoparticle-positive macrophages in dissociated hypoxic kidney organoids.

**Figure S10.** Organoid delivery and CD4⁺FOXP3⁺ T-cell frequency under normoxia. (A,B) FITC-positive organoid cells. (C,D) CD4/FOXP3 flow cytometry in the PBMC compartment.

**Figure S11.** Representative gating strategy for CD4⁺CD25⁺FOXP3⁺ cells in human kidney-organoid single-cell suspensions.

**Figure S12.** Longitudinal major-organ histology in healthy mice after intravenous D4F-CeO₂@LNP-CasRx(STING). Healthy mice were not subjected to renal pedicle clamping. They received two intravenous administrations 3 h apart at simulated time points corresponding to 2 h before clamping and 30 min after restoration of renal blood flow in the I/R protocol. The cumulative doses were 40 mg kg⁻¹ CeO₂ equivalent and 1.0 mg kg⁻¹ pDNA equivalent. Representative H&E sections from heart, liver, spleen, lung, and kidney were obtained at 5, 10, 15, 30, and 45 days (n = 3 mice per time point). This figure assesses overt histopathology under the tested regimen. Scale bar, 50 μm.

**Figure S13.** Forty-five-day hematological and biochemical assessment of D4F-CeO₂@LNP-CasRx(STING) in healthy mice. (A–D) RBC, HGB (g dL⁻¹), WBC, and PLT. (E,F) ALT and creatinine. Data are mean ± SD (n = 5 mice per group); unpaired two-tailed Student’s t-test; ns, not significant.

**Figure S14.** Representative PAS staining of kidneys from the indicated I/R groups. Scale bar, 50 μm.

**Figure S15.** Representative Masson staining of kidneys two months after I/R. Scale bar, 50 μm.

**Figure S16.** Ex vivo cytokine secretion by CD11b⁺ renal macrophages from I/R mice with or without D4F-CeO₂@LNP-CasRx(STING) treatment. (A) Isolation, culture, and ELISA workflow. (B–D) TNF-α, IL-6, and IL-10. Data are mean ± SD (n = 3 biological samples per group); unpaired two-tailed Student’s t-test; **P* < 0.05, ***P* < 0.01, and ****P* < 0.001.

**Figure S17.** Spatial maps of all six kidney sections showing the annotated cell populations in three AKI and three therapy samples.

**Figure S18.** Sample-level heatmap of the fractions of immune cells positive for selected immune-state marker genes in individual kidneys (n = 3 kidneys per group).

**Figure S19.** Dot plot of marker-gene expression used to annotate inflammatory/M1-like-enriched and pro-resolving/M2-like-enriched macrophage states and T-cell populations in the spatial transcriptomic dataset.

**Figure S20.** Pro-inflammatory/M1-like, apoptosis-associated, anti-inflammatory/M2-like, and repair/regeneration signatures along the inferred macrophage-state continuum. Pseudotime reflects computational state ordering and does not establish lineage conversion.

**Figure S21. Quantification of local immune composition in AKI and therapy kidney sections.**

**Figure S22.** Representative single gating chain for renal macrophage analysis in mouse kidneys.

**Figure S23.** Representative gating strategy for renal CD4⁺FOXP3⁺ T-cell analysis.

**Figure S24.** Predicted ligand–receptor interactions associated with condition-dependent immune-cell/proximal-tubule crosstalk. The scores represent computationally inferred interactions and do not establish direct signaling or causality.

**Figure S25.** Principal component analysis of kidney proteomes from AKI and therapy groups.

**Figure S26.** Volcano plot of differentially represented proteins between therapy and AKI samples.

**Figure S27.** Functional pathway enrichment of proteins associated with AKI or therapy.

**Figure S28.** GO functional-signature comparison between AKI and therapy groups.

**Figure S29.** Protein–protein interaction network of therapy-associated and AKI-associated modules.

**Figure S30.** Heatmaps of proteins in the STING/DNA-sensing and NF-κB pathways.

**Figure S31.** Representative injury- and repair-associated proteins, including Havcr1, Lcn2, S100a9, Lrp2, Arg1, and Mrc1.
